# Antifungal metallacarboranes modulate Hsp90 activity

**DOI:** 10.1080/17568919.2026.2732258

**Published:** 2026-09-21

**Authors:** Konrad Kubiński, Paweł Czubak, Aleksandra Martyna, Paweł Kozyra, Paulina Czaplewska, Sonja Engler, Johannes Buchner, Krzysztof Fink, Tomasz Goszczyński, Maciej Masłyk

**Affiliations:** a Department of Molecular Biology, The John Paul II Catholic University of Lublin, Lublin, Poland; b Department of Biomedical and Analytical Chemistry, The John Paul II Catholic University of Lublin, Lublin, Poland; c AI Drug Discovery Center, The John Paul II Catholic University of Lublin, Lublin, Poland; d Laboratory of Mass Spectrometry-Core Facility Laboratories, Intercollegiate Faculty of Biotechnology of UG and MUG, University of Gdansk, Gdansk, Poland; e TUM - School of Natural Sciences, Center for Protein Assemblies – CPA, Garching, Germany; f Laboratory of Biomedical Chemistry, Hirszfeld Institute of Immunology and Experimental Therapy, Polish Academy of Sciences, Wrocław, Poland

**Keywords:** Metallacarboranes, boron clusters, *Candida albicans*, Hsp90, antifungal agents

## Abstract

**Aims:**

Despite growing interest in metallacarborane biological activity, the molecular targets underlying their antifungal activity remain unknown. This study aimed to identify protein targets of antifungal metallacarborane derivatives in *Candida albicans* and to characterize their interaction with the molecular chaperone Hsp90.

**Experimental:**

Affinity chromatography using chromatographic beads bearing immobilized [CoSAN-I]^−^ was employed to pull down proteins from *C. albicans* cell lysate. Captured proteins were identified by MALDI-TOF mass spectrometry. Direct binding of [CoSAN]^−^, [CoSAN-I]^−^, and its derivatives to recombinant Hsp90 was confirmed by affinity chromatography and surface plasmon resonance. The effect of compounds on Hsp90 ATPase activity was assessed using a malachite green phosphate release assay. Molecular docking and DFT (Density Functional Theory) calculations were performed to rationalize binding mechanisms.

**Results:**

MALDI-TOF analysis identified 72 interacting proteins, of which 14 are established antifungal targets; Hsp90 showed the highest sequence coverage (82.7%). SPR confirmed direct, multivalent binding of all metallacarborane derivatives to Hsp90. The compounds modulated Hsp90 activity in a biphasic, dose-dependent manner, with maximal activation of 82–107% at 50–75 µM.

**Conclusions:**

Metallacarboranes directly engage and functionally modulate Candida albicans Hsp90, establishing a novel mechanistic basis for their antifungal activity and providing a rational framework for the development of boron-cluster-based therapeutics.

## GRAPHICAL ABSTRACT

**Figure:**
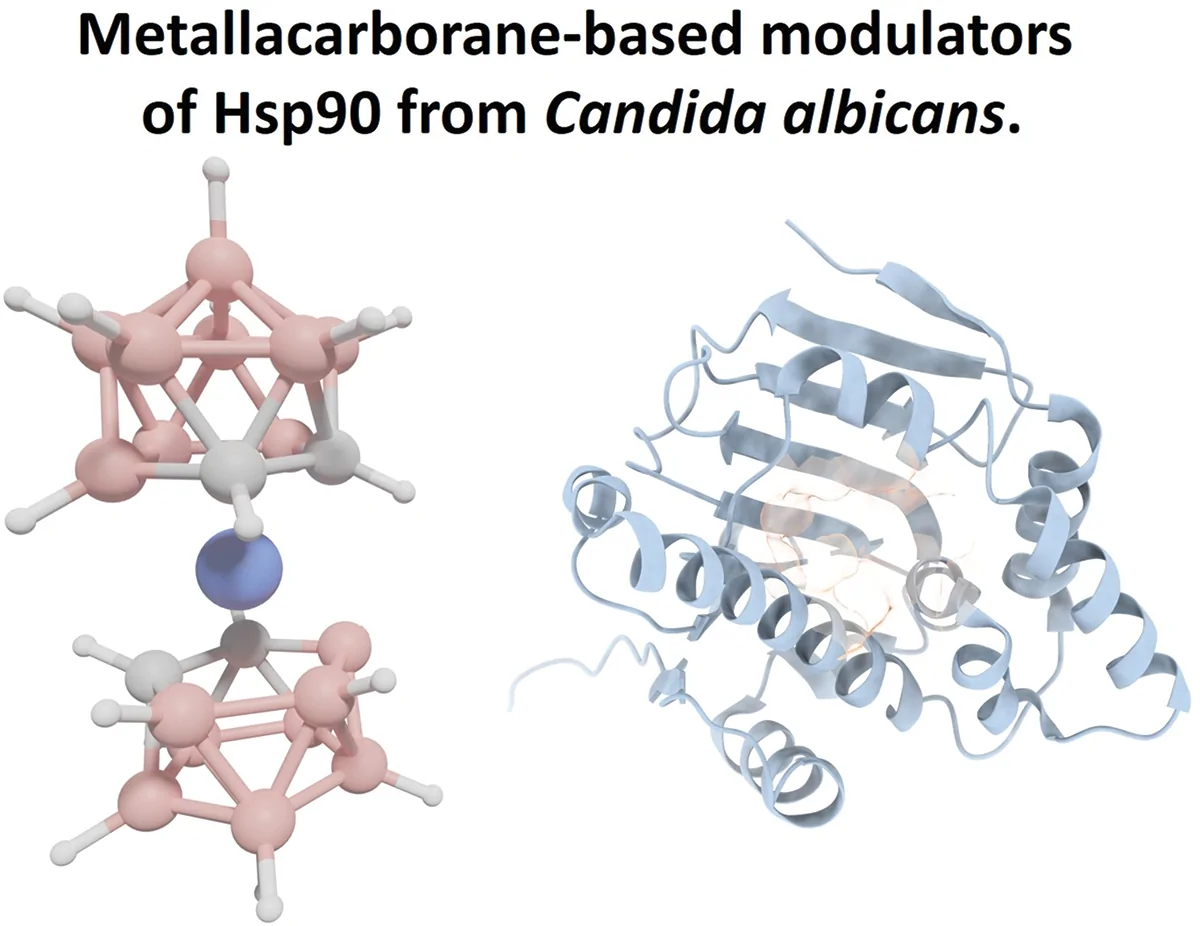

## 1. Introduction

Candidiasis, the most common opportunistic fungal disease worldwide, is caused by over 20 Candida species. Among them, *Candida albicans* remains the most frequently isolated pathogen, accounting for approximately 50–70% of human candidiasis cases, depending on the clinical setting (e.g., mucosal vs. systemic), geographic region, and the patient’s immune status [1–3]. These infections significantly contribute to global morbidity and mortality; in particular, invasive candidiasis (candidemia) is considered a life-threatening condition, with mortality rates ranging from 30% to 50% in critically ill and immunocompromised patients [4,5]. Unfortunately, the pathogenesis of Candida species remains incompletely understood, overall infection rates are rising globally (exceeding hundreds of thousands of life-threatening cases annually), and resistance to systemic antifungal drugs – such as azoles and echinocandins–is increasingly reported [6,7]. Therefore, early diagnosis and developing novel therapeutic strategies are crucial to fight this pathogen.

Referring to the above-mentioned issues, a promising solution appeared in compounds containing abiotic motifs like metallacarboranes. These are organometallic species defined by a polyhedral cage structure containing boron and carbon atoms, with metal atoms embedded within or between carborane cages. In many cases, two carborane ligands sandwiching a metal center – such as in bis-(dicarbollides) - give rise to distinctive chemical properties. These sandwich architectures contribute to extraordinary chemical properties, opening up diverse potential applications [8–11]. The most extensively studied metallacarborane contains a cobalt(III) cation, specifically known as 3-cobalt bis(1,2-dicarbollide) ([3,3’-Co(1,2-C_2_B_9_H_11_)_2_]^−^) and abbreviated as [CoSAN]^−^. This 3D aromatic anion is characterized by thermal, chemical, and biological stability, along with good solubility in both organic and aqueous solvents, depending on the counterion [9,12–14]. It exhibits nonclassical amphiphilic behavior, partially due to the delocalization of its anionic charge throughout its volume, resulting in a low charge density. [CoSAN]^−^ forms strong dihydrogen bonds, facilitating interactions with amino acids and proteins [15,16]. In biological environments, it can cross lipid membranes without disrupting their integrity and demonstrates low toxicity both *in vitro* and *in vivo* [9,13]. The abiotic nature of these systems is particularly significant, as metallacarboranes are foreign to biological systems, enzymatic pathways, and living organisms in general, making them resistant to catabolism - a desirable trait for therapeutic applications. This is especially important when developing new antibiotics.

To date, some biological activities of metallacarboranes have been published, including anticancer, anti-viral, and anti-microbial [10,17,18]. These compounds can also act as carriers for membrane-impermeable cargos, facilitating the transport of therapeutic agents into cells without compromising its membrane integrity [19,20]. Metallacarboranes have demonstrated significant antimicrobial properties against a variety of bacterial and fungal species [21–24]. Their efficacy is attributed to the unique structure and chemical properties of these compounds, which allow them to interact with microbial cell membranes and disrupt cellular functions. In 2022, Kubiński et al. reported a new class of boron clusters as effective anti-*C. albicans* agents [21]. The compounds presented high activity, limited toxicity, and the ability to overcome resistance to systemic drugs when tested against clinical strains. It is worth mentioning that overcoming the drug resistance mechanisms developed by microorganisms is crucial for creating effective antifungal therapies, offering hope for successfully treating infections that are otherwise difficult to cure. Unfortunately, the mechanisms of metallacarborane activity in cells are not well understood. Identifying molecular targets for metallacarboranes is crucial, as it allows the rational development of new derivatives that achieve optimal properties and enhanced biological efficacy.

Effective antifungal therapy relies on targeting proteins and cellular structures essential for the survival, virulence, and pathogenicity of Candida species. Current antifungal agents exploit a limited repertoire of well-validated targets: ergosterol biosynthesis enzymes (azoles), β-1,3-D-glucan synthase (echinocandins), thymidylate synthase and RNA incorporation (flucytosine), and chitin synthesis enzymes (nikkomycin Z) [25]. However, the rising prevalence of drug resistance and the restricted target space underscore the urgent need to identify novel druggable proteins [6]. The virulence of *C. albicans* is governed by a network of interconnected processes, including morphogenetic switching between yeast and hyphal forms, biofilm formation, stress response, and metabolic adaptation to host-imposed conditions [26]. Among the molecular regulators of these processes, the ATP-dependent molecular chaperone Hsp90 occupies a uniquely central position: it stabilizes key effectors of antifungal resistance mechanisms (such as calcineurin) and governs temperature-dependent fungal morphogenesis, making it a particularly attractive target for antifungal drug development. Hsp90 experimental inhibitors, such as geldanamycin and radicicol, have demonstrated antifungal potential, further validating this target [25,27]. Identifying novel compounds capable of engaging Hsp90 and other virulence-related proteins in Candida thus represents a highly relevant and rational strategy.

With regard to metallacarborane–protein interactions, data in the literature remain extremely limited. Nevertheless, specific interactions have been documented: derivatives of [CoSAN]^−^ have been shown to act as potent and selective inhibitors of HIV-1 protease [28] and of carbonic anhydrase IX (CA IX), a tumor-associated enzyme, where the bulky and hydrophobic [CoSAN]^−^ cage enables efficient, multivalent binding within the enzyme’s active site, yielding high inhibitory potency and selectivity [29]. Additionally, strong yet nonspecific interactions have been observed with serum albumin and myoglobin, where [CoSAN]^−^ - in contrast to classical chaotropes (which often denature proteins) - induces reversible protein self-assembly [15,16,30].

Hsp90 functions as a master regulator of multiple virulence-associated processes in *C. albicans*. It stabilizes key signal transducers - most notably the protein phosphatase calcineurin and Mkc1 - enabling cellular adaptation to membrane- and cell wall-active antifungal drugs [31,32]. Hsp90 also governs the yeast-to-hypha morphogenetic transition critical for tissue invasion, through temperature-dependent repression of Ras1–cAMP–PKA signaling [33,34]. In the context of biofilm formation, Hsp90 depletion impairs *C. albicans* biofilm maturation and dispersal, and abrogates azole resistance of biofilm cells both *in vitro* and *in vivo* [35]. Finally, by stabilizing calcineurin, Hsp90 mediates tolerance to both azoles and echinocandins; its inhibition transforms fluconazole from ineffective to highly active against resistant isolates [36]. The breadth of Hsp90‘s influence on *C. albicans* pathobiology - spanning stress adaptation, morphogenesis, biofilm integrity, and antifungal resistance - makes it a particularly compelling therapeutic target.

In this study, we chose highly potent metallacarborane derivatives (Figure 1(a)) (active against reference and resistant *C. albicans* isolates) and probed their molecular protein targets. We fished out proteins from a *C. albicans* cell lysate using beads carrying immobilized [CoSAN-I]^−^, identified them via MALDI-TOF, and focused in particular on the interaction with the molecular chaperone Hsp-90 - seen as a promising antifungal target [37]. Hsp90 is the central part of a complex chaperone machinery in the cytosol of eukaryotes which is required for the folding of hundreds of different client proteins [38]. ATP binding and hydrolysis by Hsp90 are important for client processing [39].

**Figure 1.**
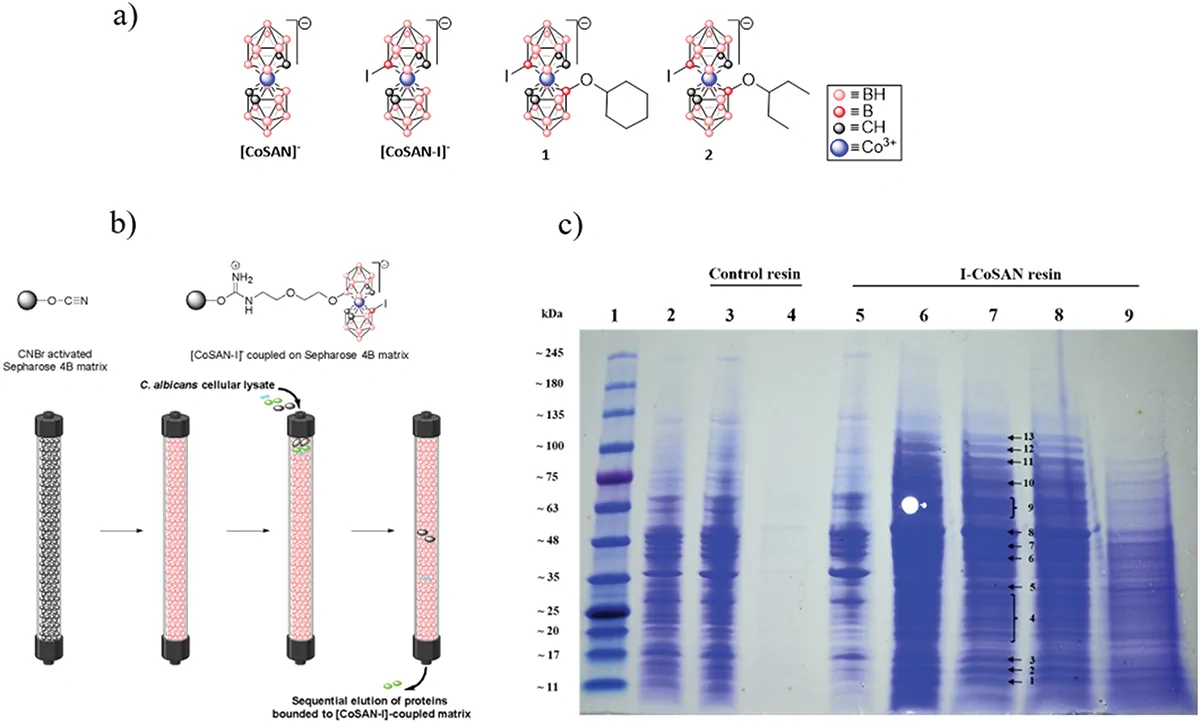
(a) Structures of metallacarborane derivatives active against both reference and drug-resistant *Candida albicans* isolates, selected for this study; (b) Fishing out proteins from a *C. albicans* cell lysate using beads bearing immobilized [CoSAN-I]^−^. (c) SDS-PAGE result of an affinity chromatography of *C. albicans* lysate using control and [CoSAN-I]^−^ resin. 1 – Perfect TM Tricolor Protein Ladder (EURx), 2 – *C. albicans* cellular lysate, 3 – fraction of proteins not bound to the control resin (flow-through), 4 – fraction of proteins eluted from the control resin after 0.15 M NaCl wash, 5 – fraction of proteins not bound to the I-[CoSAN]^−^ resin (flow-through), 6 – fraction of proteins eluted from the [CoSAN-I]^−^ resin after 0.15 M NaCl wash, 7 – fraction of proteins eluted from the [CoSAN-I]^−^ resin after 0.5 M NaCl wash, 8 – fraction of proteins eluted from the [CoSAN-I]^−^ resin after 1 M NaCl wash, 9 – fraction of proteins eluted from the [CoSAN-I]^−^ resin after 2 M NaCl wash. Numbered arrows and braces in lane 7 indicate protein bands selected and excised from the gel for further MALDI‑TOF analysis.

We confirmed the physical interaction between derivatives of [CoSAN]^−^ and Hsp90 using affinity chromatography as well as surface plasmon resonance (SPR), and discovered an influence of the compounds on the ATPase activity of Hsp90, which was supported by molecular docking calculations.

## 2. Experimental

### 2.1. Synthesis

Compounds **1** and **2** were synthesized in our laboratory, and the procedures along with analytical data were published recently and are described below [40]. Compounds used for immobilization on Sepharose-4B (Figure S1) were synthesized following published procedures described below [22,40–43].

Cs[CoSAN], cesium [3,3’-cobalt bis(1,2-dicarbollide)], was purchased from Katchem (Prague, Czech Republic).

#### 2.1.1. Synthesis of substrates

**Cs[CoSAN-I]** was synthesized according to literature methods [44]. Briefly, Iodine (3.63 g, 14.3 mmol) was added to a solution of Cs[CoSAN] (3.34 g, 7.31 mmol) in ethanol (40 mL). The reaction mixture was left to stand overnight at room temperature and was then heated under reflux for 2.5 h. Excess iodine was quenched by addition of an aqueous solution of Na_2_SO_3_ (1.48 g, 11.7 mmol in 32 mL of water). The mixture was concentrated to approximately half of its initial volume and left to crystallize. The resulting crystals were filtered off, washed with water, and dried in vacuo to afford the product (3.87 g, 91%).

**[8,8’-µ-I-3,3’-Co(1,2-C2B9H10)2] (bridged iodonium derivative)**. The synthesis was performed according to the previously described method [40]. Briefly, AlCl3 (206 mg, 1.54 mmol) was added to a suspension of Cs[CoSAN-I] (300 mg, 0.515 mmol) in anhydrous toluene (70 mL), and the reaction mixture was stirred at room temperature for 20 h under an argon atmosphere. The insoluble residue was removed by filtration, and the filtrate was combined with silica gel (0.4 g) and stirred for 5 min. The mixture was filtered, and the solvent was removed under reduced pressure. The resulting red solid was dried under vacuum for 24 h to afford the product (220 mg, 95%).

**[3,3’-Co(8-C4H8O2-1,2-C2B9H10)(1,’2’-C2B9H11)] (CoSAN] cyclic oxonium derivative)**. The synthesis was performed according to the previously described method [41]. Briefly, Cs[CoSAN] (890 mg, 1.95 mmol) was suspended in 1,4-dioxane (100 mL), and BF_3_·Et_2_O (2.0 mL, 16.0 mmol) was added. The reaction mixture was heated under reflux for 5 h under an argon atmosphere. After cooling to room temperature, the mixture was filtered, and the solvent was removed under reduced pressure. The residue was dissolved in dichloromethane and passed through a short silica gel pad using dichloromethane as the eluent. The eluate was concentrated in vacuo to afford the product (0.750 g 94%).

**[3,3’-Co(8-C4H8O2-1,2-C2B9H10)(8’-I-1,’2’-C2B9H10)]**, [CoSAN-I] cyclic oxonium derivative. The synthesis was performed according to the previously described method [22]. Briefly, a suspension of the [CoSAN]-bridged iodonium derivative (300 mg) in anhydrous cyclohexane (40 mL) was treated with 1,4-dioxane (1 mL), homogenized in an ultrasonic bath, and heated under reflux for 60 min. After cooling to room temperature, the orange precipitate was isolated by centrifugation, washed with cyclohexane (10 mL) and petroleum ether, and dried under vacuum to afford the product (315 mg, 88%).

#### 2.1.2. Synthesis of compounds for biological evaluation (Figure S1(A))

**Cs[3,3’-Co(8-O-cycloC6H11-1,2-C2B9H10)(8’-I-1,’2’-C2B9H10)] (1)** The synthesis was performed according to the previously described method [40]. Briefly, the [CoSAN]-bridged iodonium derivative (98 mg, 0.22 mmol) was dissolved in anhydrous cyclohexanol (10 mL), and the reaction mixture was left to stand at room temperature for 20 h under an argon atmosphere. The solvent was removed under reduced pressure, and the resulting yellow solid was dried under vacuum. The crude product was dissolved in acetone (8 mL), and an aqueous solution of CsCl (300 mg in 8 mL of cold water) was added. The mixture was concentrated until precipitation of an orange solid occurred. The precipitate was isolated by centrifugation, washed with cold water (3 mL), and dried under vacuum to afford the product (119 mg, 80%).

**Cs[3,3’-Co(8-O-CHEt2-1,2-C2B9H10)(8’-I-1,’2’-C2B9H10)] (2)** The synthesis was performed according to the previously described method [40]. Briefly, the [CoSAN-I]-bridged iodonium derivative (103 mg, 0.23 mmol) was dissolved in anhydrous 3-pentanol (10 mL), and the reaction mixture was left to stand at 40°C for 20 h. The subsequent work-up procedure was identical to that described for compound **1**. The product was obtained in 77% yield (118 mg).

#### 2.1.3. Synthesis of compounds for immobilization on sepharose-4B (Figure S1(B))

**[3,3’-Co(8-OCH2CH2OCH2CH2-NH3-1,2-C2B9H10)(1,’2’-C2B9H11)] (3)**. The synthesis was performed according to the previously described method [43]. Briefly, a suspension of the cyclic oxonium derivative of [CoSAN] (90 mg, 0.22 mmol) in acetonitrile (10 mL) was treated with aqueous ammonia (25%, 1 mL) and stirred overnight at room temperature. The solvent was removed under reduced pressure, and the resulting residue was dried under vacuum. The crude oily product was dissolved in methanol (5 mL) and treated with 1 M HCl(aq) (20 mL). The resulting red oil was isolated by centrifugation, and the supernatant was discarded. The product was washed with cold water (5 mL) and freeze-dried to afford the final compound (77% yield, 72 mg).

**[3,3’-Co(8-OCH2CH2OCH2CH2-NH3-1,2-C2B9H10)(8’-I-1,’2’-C2B9H11)] (4)**. The synthesis was performed according to the previously described method [42]. Briefly, a suspension of the cyclic oxonium derivative of [CoSAN-I] (106 mg, 0.20 mmol) in acetonitrile (10 mL) was treated with aqueous ammonia (25%, 1 mL) and stirred overnight at room temperature. The subsequent work-up procedure was identical to that described for compound **3** (66% yield, 72 mg).

### 2.2. Immobilization of metallacarborane molecule on chromatographic beads

CNBr-activated Sepharose 4B, 1.0 g of dry powder, was allowed to swell in 35 mL of cold 1 mM HCl. The swollen gel was washed with coupling buffer, 0.1 M NaHCO_3_, pH 8.3, and then suspended in the same buffer containing the appropriate amount of the amine-functionalized [CoSAN]^−^ or [CoSAN-I]^−^ derivative, Figure S1. The suspension was incubated under gentle mixing to allow covalent coupling of the metallacarborane ligand to the activated matrix. After completion of the coupling reaction, the resin was separated from the supernatant and washed extensively with coupling buffer. The supernatants and washing fractions were collected, and the residual nonimmobilized [CoSAN]^−^ or [CoSAN-I]^−^ amine derivative was quantified by HPLC (High-Pressure Liquid Chromatography). The immobilized ligand density was calculated by difference between the amount of compound initially added and the amount recovered in the supernatant and washing fractions. Ligand loadings of 1.8 and 2.0 μmol per mL of drained gel were obtained for the [CoSAN]^−^- and [CoSAN-I]^−^-modified Sepharose 4B materials, respectively. Residual active groups were blocked by treatment with an ethanolamine-containing blocking buffer, and the resulting metallacarborane-functionalized Sepharose 4B beads were washed thoroughly prior to use.

To assess possible noncovalent adsorption of metallacarboranes to CNBr-activated Sepharose 4B, control experiments were performed under the same conditions using the corresponding non-aminated cesium salts, Cs[CoSAN] and Cs[CoSAN-I]. In both cases, HPLC analysis of the supernatants and washing fractions showed quantitative recovery of the metallacarborane species, indicating no detectable noncovalent binding to the Sepharose matrix. The functional performance of the metallacarborane-modified beads was evaluated using bovine serum albumin, BSA (Bovine Serum Albumin), as a model protein, because strong interactions between BSA and COSAN derivatives have been reported previously [45].

### 2.3. Affinity chromatography and SDS‑PAGE electrophoresis

*C. albicans* ATCC 10231 was used as the reference strain throughout this study. For preparation of cell lysates, a single colony was inoculated into Sabouraud Dextrose Broth (SDB; Merck, Germany; composition: 10 g/L peptone, 40 g/L dextrose, pH 5.6 ± 0.2) and cultured overnight at 37°C with orbital shaking at 200 rpm to generate a pre-culture. The pre-culture was diluted 1:50 (v/v) into fresh SDB and grown at 37°C, 200 rpm for approximately 3–4 h. Exponential growth phase was defined as an optical density at 600 nm (OD_600_) of 0.4–0.6, corresponding to approximately 1–5 × 10^6^ CFU/mL, as confirmed by colony-forming unit counting on SDA plates. Cells were harvested by centrifugation (3,000 × g, 10 min, 4°C), washed twice with ice-cold phosphate-buffered saline (PBS, pH 7.4), and immediately processed for lysate preparation. Cells were disintegrated using bead beating with glass beads in 10 20-second cycles with cooling in an ice bath between cycles. 220 µl of the resulting lysate with a protein concentration of 23 mg/ml was mixed with 60 µl of chromatographic resin containing immobilized [CoSAN-I]^−^ ligand. A control experiment was simultaneously conducted using sole resin. The mixture of lysate and resin was incubated for 1 hour in an ice bath with gentle shaking on a laboratory rocker. The resin was then gently centrifuged and washed multiple times using four sequential solutions with increasing NaCl concentrations of 150 mM, 500 mM, 1000 mM, and 2000 mM. Bound proteins were eluted from the resin by adding 60 µl of SDS-PAGE buffer (125 mM Tris-HCl, pH 6.8; 20% glycerol; 3% DTT; 4% SDS; 0.001% bromophenol blue). Samples were incubated at 95°C, then centrifuged, and the supernatant was subjected to SDS-PAGE on a 4–15% gradient acrylamide gel (Bio-Rad). After the separation was complete, protein bands were stained with Coomassie blue. Selected visible protein bands were then excised from the gel and each placed separately in a tube containing 1% acetic acid. Such prepared samples were subjected for mass spectrometry analysis to identify the proteins contained in the bands.

### 2.4. MALDI-TOF/TOF mass spectrometry

Spots of interest were cut from the gels and placed in separate Eppendorf tubes. In-gel digestion was performed according to standard protocol consisting of gel discoloration and removal of Coomassie staining, reduction and alkylation with dithiothreitol (DTT) and iodoacetamide (IAA) respectively. Digestion was carried out overnight with trypsin (Promega Mass Spectrometry Grade Gold) at 37°C. Then, the obtained tryptic peptides were eluted from the gel spots into the solution with sequential washing of gel pieces with 50 mM ammonium bicarbonate buffer, 5% formic acid in 50% acetonitrile and 100% acetonitrile [46]. Next all samples were concentrated (SpeedVac) and the final cleanup was carried out using the Zip Tip method. Briefly, using Zip Tips (Millipore) packed with the C18 resin, samples were repeatedly aspirated and dispensed to facilitate analyte binding, then washed to remove impurities, and finally eluted with 50% acetonitrile containing 0.1% formic acid, yielding material suitable for further mass spectrometry analysis.

ESI LC-MS/MS analysis was performed on Triple Tof 5600+ mass spectrometer with DuoSpray Ion Source (AB SCIEX, Framingham, MA) connected to the Eksigent microLC (Ekspert MicroLC 200 Plus System, Eksigent, Redwood City, CA) equipped with the ChromXP C18CL column (3 μm, 120 Å, 150 × 0.5 mm). The microLC−MS/MS system was controlled by the AB SCIEX Analyst TF 1.6 software. Chromatographic separation was carried out for 50 min in a gradient program: (1) 0 − 2 min − 3.5% solvent B, (2) 2 − 50 min − 3.5 − 20% solvent B, (3) 50 − 54 min −98% solvent B, (4) 54–56 min −98% solvent B, and (5) 56.1–60 3.5% B where solvent A was 0.1% formic acid in water and solvent B 0.1% formic acid in acetonitrile. The identification of proteins present in the examined gel bands was carried out based on the obtained fragmentation spectra using the ProtinPilot software (v 4.5) and the appropriate protein database (Uniprot *C. albicans*) with an automated false discovery rate (1% FDR (False Discovery Rate)) [47].

### 2.5. Hsp90 binding to immobilized [I-CoSAN]^−^

Due to the unavailability of the Hsp90 homolog from *C. albicans*, a protein identical by 81.52% and similar by 92%, Hsp90 from *Saccharomyces cerevisiae* (P02829, UniProt), was used. Recombinant yeast Hsp90 was produced and purified as previously described [48]. Briefly, N-terminal 6x-His-tagged yeast Hsp90 (Hsp82) was expressed in *E. coli BL21*. Cells were sonicated in phosphate buffer (pH 7.8) containing 300 mM NaCl, 10 mM imidazole, DTT, protease inhibitors, and DNaseI. Cleared lysates (40,000 × g, 1 h, 6°C) were purified on a His-Trap FF column, eluting with 500 mM imidazole. The diluted eluate was subjected to anion exchange chromatography (RESOURCE Q) using a KCl gradient. Pooled and concentrated fractions (30 kDa MWCO) were polished via size-exclusion chromatography (Superdex 200 pg) in 40 mM HEPES, 150 mM KCl, and 5 mM MgCl_2_. Protein purity was confirmed by SDS-PAGE before flash-freezing. 5 µg of recombinant non-tagged Hsp90 protein was added to 50 µl of chromatographic beads containing immobilized [CoSAN-I]^−^ ligand and chromatographic beads without ligand (control). Then, PBS (phosphate‑buffered saline) was added to 200 µl, and the mixtures were incubated for 1 hour at 4°C with agitation. After this time, the samples were centrifuged, and the supernatants (flow-through, FT) were collected. Next, the beads were washed five times with a PBS buffer containing 150 mM NaCl and centrifuged. The supernatants from the first and final centrifugation were collected. Afterward additional washings, with the use of a PBS buffer containing 300 and 500 mM NaCl, were conducted and following the centrifugation, supernatants were collected.

After washing, 50 µl of SDS-PAGE buffer (125 mM Tris-HCl, pH 6.8; 20% glycerol; 3% DTT; 4% SDS; 0.001% bromophenol blue) was added to the beads. Samples were incubated at 95°C to elute the Hsp90 protein, then centrifuged. The collected FT samples, washing samples, and elution samples were subjected to SDS-PAGE electrophoresis.

### 2.6. Influence of metallacarboranes on Hsp90 activity

ATPase activity of Hsp90 was assessed using the ADP-Glo assay (Promega), which measures the amount of ADP produced during ATP hydrolysis. The assay was performed according to the manufacturer’s protocol, and luminescence was recorded as a readout of enzymatic activity. To evaluate potential inhibitory effects on protein Hsp90, [CoSAN]^−^, [CoSAN-I]^−^, and **1** were tested individually at concentrations ranging from 0 to 200 µM. Each compound was incubated with Hsp90 under assay conditions. Luminescence was measured using a Synergy H1 microplate reader (BioTek).

### 2.7. Surface plasmon resonance (SPR) analysis

Surface plasmon resonance (SPR) experiments were carried out using HBS-N buffer (10 mM HEPES, pH 7.4, containing 150 mM NaCl). The buffer was filtered through a 0.2 µm membrane and degassed before use.

#### 2.7.1. Preparation of samples

Hsp90 was initially prepared in 40 mM HEPES buffer, pH 7.5 with 150 mM KCl, 5 mM MgCl_2_, and 2 mM dithiotreitol (DTT) at 230 µM. Before immobilization, the solution was diluted tenfold in 10 mM HEPES buffer, pH 7.4, containing 150 mM NaCl and 0.25 mM tris(2-carboxyethyl)phosphine (TCEP), and dialyzed against the same buffer. Radicicol was dissolved in DMSO (dimethyl sulfoxide) at 4 mM. Cs[CoSAN]^−^ and Cs[CoSAN-I]^−^ were dissolved in DMSO at 100 mM. **1** and **2** were dissolved in DMSO at 40 mM.

#### 2.7.2. Immobilization of Hsp90

Immobilization was conducted at 25°C using a CM5 sensor chip and standard Biacore^TM^ reagents (GE Healthcare Life Sciences). The surface was activated with a solution of 0.2 M N-ethyl-N’-(3-dimethylaminopropyl)carbodiimide and 50 mM N-hydroxysuccinimide for 7 min. Hsp90 (23 µM in 10 mM HEPES buffer, pH 7.4, with 150 mM NaCl and 0.25 mM TCEP) was diluted to 2.3 µM in 10 mM sodium acetate (pH 5.0) and injected for 800 s at 5 µl/min. Residual reactive groups were blocked with 1 M ethanoloamine (pH 8.5). Noncovalently bound protein was removed and the baseline stabilized by two 10-s injections of 50 mM NaOH. The final immobilization level was 8.6 kRU. The reference channel underwent the same procedure without protein injection.

#### 2.7.3. Kinetic measurements

Analytes were injected over the reference channel and the Hsp90-coated channel for 60 s at a flow rate of 30 µl/min. Each cycle included a 60-s baseline stabilization, a 60-s injection, and a 600-s dissociation phase. Buffer-only injections were included for double referencing. Radicicol was tested at 0.05–0.5 µM, while [CoSAN]^−^, [CoSAN-I]^−^, **1**, and **2** were tested at 0.5–5.0 µM. All analytes contained 0.5% DMSO and were injected in triplicate.

#### 2.7.4. Analysis

Sensorgrams for the reference channel were subtracted from sensorgrams for the channel with Hsp90. Subsequently, sensorgrams of the buffer containing 0.5% DMSO were subtracted from sensorgrams of the compounds. Kinetic constants (k_on_ and k_off_) were determined by fitting sensorgrams to a 1:1 Langmuir binding model using Biacore Evaluation Software 1.0. The dissociation constant (K_D_) was calculated by the equation K_D_ = k_off_/k_on_.

### 2.8. Computational chemistry details

#### 2.8.1. Geometry optimization

The tested compounds ([CoSAN]^−^, [CoSAN-I]^−^, **1**) were sketched using Spartan “20” [49], and then their geometry was preliminarily optimized using a molecular mechanics force field (MMFF94) in the ground state in gas. Quantum chemical calculations were performed using density functional theory (DFT) implemented in the ORCA version 6.0 software package [50,51]. The exchange-correlation functional was evaluated using the LibXC library, version 7.0.0 [52]. The geometries of the studied metallacarborane complexes were fully optimized without symmetry constraints. The geometry and electronic properties of the compound were investigated for its open-shell anionic state, corresponding to a total charge of −1 and a spin multiplicity of **1**. To ensure the accuracy of the electronic structure description, which is particularly important for describing noncovalent interactions and dispersion forces often found in metallacarboranes, the hybrid functional ωB97M-V with range separation and nonlocal correlation (VV10) was used [53,54]. The calculations utilized the def2-TZVP (Triple-Zeta Valence Polarized) basis set for all atoms [55], which provides a balanced description of the valence and core orbitals, essential for boron-rich clusters and metal centers. To accelerate the calculations, the Resolution of Identity (RI) approximation for the Coulomb integrals was used along with the matching def2/J auxiliary basis set [56,57]. To account for solvent effects relevant to the biological evaluation environment, an implicit solvation model (CPCM) was used, using dimethyl sulfoxide (DMSO) as the solvent [58]. Accuracy of numerical integration was ensured by setting the grid quality to DefGrid3, and the self-consistent field (SCF) convergence criteria were tightened using the TightSCF keyword to minimize numerical noise.

#### 2.8.2. Wavefunction analysis and visualization

Post-processing of the electronic structure data was facilitated by converting the ORCA output files to the Molden format. The **Multiwfn 3.8** software package was employed for the detailed topological analysis of the wavefunction [59,60]. Specifically, the Frontier Molecular Orbitals (FMOs), including the Highest Occupied Molecular Orbital (HOMO) and the Lowest Unoccupied Molecular Orbital (LUMO), were computed and subsequently visualized using **VMD 2.0.0a5** [61]. Molecular Electrostatic Potential (MEP) was mapped onto the electron density isosurfaces and visualized using the **Avogadro 1.102.1** [62].

#### 2.8.3. Molecular docking

The structure of the *C. albicans* Hsp90 nucleotide binding domain in complex with AMPPNP (PDB code 9MTB [63]) was selected as the molecular target. Hydrogen atoms were added and the protein was loaded according to the procedure [64]. This involves loading the protein as a “macromolecule” into the program, which automatically adds charges, merges nonpolar hydrogens, and assigns appropriate atom types. Autodock Tools v.1.5.7 and AutoDock-4.2.6 software were used for molecular docking [65]. Ligands were also prepared using the same method [64], which involves defining the center of the torsional tree and selecting torsional degrees of freedom. The ligands were loaded using default settings. The cobalt charge was manually modified to 3+ in the pdbqt files. Molecular docking was performed using the Lamarckian genetic algorithm [66], with GA runs set to 50 and the remaining parameters assumed as default. The protein was treated as rigid. A grid box of 34 × 18 × 22 points with a spacing of 0.375 Å was defined, centering the ligand at the active site (gridcenter coordinates x = 15.512, y = −3.52, z = −3.351). The presented positions of the docked ligands were selected based on the scoring function and visual inspection.

To verify the molecular docking parameters, the root mean square deviation (RMSD) of the atom pairs of the compound re-docked with the native protein ligand (PDB code 9MTB [63]) was calculated using Maestro 14.5.160 [67]. Positions with an RMSD of 1.55 Å were obtained, indicating the correct choice of molecular docking method and its high precision.

Then, cross-validation was performed using the crystal structure of HIV protease with metallacarborane complex [28]. [CoSAN]^−^ was selected as the ligand. Ligand and protein preparation was the same as described above. The remainder of the method is the same as described above, using the same parameters, only changing the grid center coordinates (x = 39.968, y = −0.038, z = 5.645). A superimposition of our compound ([CoSAN]^−^) relative to the crystallographic coordinates of the native ligand is shown in Figure S2. The compounds dock at almost the same location, indicating a good choice of docking method.

ChimeraX was used to generate a molecular representation of each protein-ligand complex [68].

## 3. Results and discussion

### 3.1. Immobilization of the metallacarborane molecule [CoSAN-I]^−^ on chromatographic beads

Our goal was to discover a protein target of the antifungal activity of metallacarboranes. [CoSAN]^−^ was a starting molecule for the synthesis of a series of antifungal metallacarborane derivatives with MIC values of 120 µg/ml and 15–120 µg/ml against *C. albicans* and other *Candida* species, respectively [21]. The first derivative, [CoSAN-I]^−^ possesses an additional iodine atom and shows higher antifungal activity with MIC values of 15 µg/ml and 3.7–15 µg/ml against *C. albicans* and non-albicans *Candida* species, respectively. Since the molecular basis of the antifungal activity of metallacarboranes is almost *terra incognita*, we decided to study whether the iodine derivative of [CoSAN]^−^ can interact with the *C. albicans* proteome. For this purpose, we chose an affinity chromatography method using chromatographic beads with immobilized [CoSAN-I]^−^ (Figure 1(b)).

### 3.2. Interaction of proteins from *Candida albicans* with immobilized metallacarboranes and their identification

The lysate from the reference strain of *C. albicans* was mixed with the above-described beads. After protein binding, [CoSAN-I]^−^-resin was washed using a stepwise gradient of NaCl (0.15–2 M). After each wash step proteins were eluted with a SDS-PAGE sample buffer and resolved by SDS-PAGE (Figure 1(c)). Each sample for MALDI‑TOF analysis contained 1–3 protein bands excised from lane 7, as shown in Figure 1(c). The experimental control consisted of a sample in which *C. albicans* lysate was combined with chromatographic resin lacking the immobilized ligand ([CoSAN-I]^−^). As shown in lane 4 of Figure 1(c), only trace amounts of protein bound to the control resin.

MALDI TOF analysis of the spots revealed 1,168 recurring records, corresponding to different peptides that can be attributed to specific proteins. Further analysis of the obtained results indicated the presence of peptide fragments from 239 proteins in the analyzed bands excised from the gel. To exclude non-relevant records and limit the number of proteins for further analysis, we assumed a threshold of 50% coverage for the match between the revealed amino acid sequences and the protein sequences available in the UniProt database. This operation reduced the number of proteins of interest to 72 (Table S1).

The proteins listed in Table S1 show various cellular localizations as well as cellular functions and the majority of them are directly engaged in neither pathogenicity nor drug resistance of *C. albicans*. Functional annotation of the 72 proteins identified in the exploratory affinity-capture screen indicated representation of proteins involved in core cellular processes, including translation, energy metabolism, proteostasis, membrane homeostasis, and stress responses. Several identified proteins (Table 1) have previously been implicated in fungal virulence, stress adaptation, antifungal drug tolerance, or the mechanisms of action of antifungal agents. Because this gel-based screen was not designed as replicated quantitative proteomics, these functional annotations are interpreted as hypothesis-generating rather than as evidence of selective pathway enrichment.

**Table 1.** Selected proteins captured by [CoSAN-I]^−^ affinity resin with relevance to fungal virulence or antifungal drug action.

Protein name	Biological function	Role in pathogenicity/infection	Species/model	Citation
Alpha,alpha-trehalose-phosphate synthase	Trehalose biosynthesis – stress protection	Supports fungal survival under stress conditions	N.d.	[63]
ATP synthase subunit beta	Mitochondrial ATP synthesis	Essential for energy metabolism	*Candida glabrata*	[64]
Elongation factor 1-alpha	Translation elongation	Protein synthesis	*Aspergillus fumigatus*	[65]
EF 1-alpha 1	Translation elongation	Involved in translation elongation	*Saccharomyces cerevisiae*	[66]
EF 1-alpha 2	Translation elongation	Involved in translation elongation	*Saccharomyces cerevisiae*	[66]
Elongation factor 2	Translation elongation	Functionally essential elongation factor; historically validated as a toxin-sensitive translation target	*Saccharomyces cerevisiae*	[67,68]
FK506-binding protein 1	Regulator of calcineurin pathway	Targeted by FK506, involved in stress signaling	*Candida albicans*	[31]
Hsp90 homolog	Molecular chaperone	Stabilizes antifungal resistance mechanisms	*Candida albicans*	[69]
Hsp SSA1	Hsp70 family chaperone	Supports fungal adaptation to host-imposed stress; potential target for combinatorial antifungal therapy	*Candida albicans*	[70,71]
Hsp SSA2	Hsp70 family chaperone	Supports fungal adaptation to host-imposed stress; potential target for combinatorial antifungal therapy	*Candida albicans*	[70]
HMG-CoA synthase	Ergosterol biosynthesis pathway	Targeted in antifungal strategies via modulation of ergosterol biosynthesis; relevant for resistance management	*Candida albicans*	[72]
Phosphoglycerate kinase	Glycolysis	Conserved ATP-generating enzyme; potential antifungal target due to its role in fungal survival	N.d.	[73]
Pyruvate decarboxylase	Anaerobic fermentation	Crucial under hypoxic conditions in host tissues	*C. albicans*	[74]
V-type ATPase subunit A	pH homeostasis, vacuolar function	Essential for virulence and growth	*C. albicans*	[75]

Many proteins listed are involved in fundamental processes crucial for fungal survival and virulence, such as translation elongation, ATP synthesis, ergosterol biosynthesis, glycolysis, or pH homeostasis. Several proteins are highlighted for their roles in stress response (e.g., alpha, alpha-trehalose-phosphate synthase, Hsp family chaperones), virulence (e.g., V-type ATPase subunit A) and antifungal resistance (e.g., Hsp90 homolog). Finally, some of them are mentioned as proteins that are targets of existing antifungal compounds (FK506-binding protein 1, HMG-CoA synthase).

Since our studies were focused on molecular mechanisms of metallacarborane antifungal action, among the proteins identified in the CoSAN-I affinity-capture eluate, the ATP-dependent molecular chaperone Hsp82 (C4YTQ8, UniProt), known also as heat shock protein 90 homolog (P46598, UniProt) was selected for follow-up studies because it was detected in a gel band (Figure 1c, band 11, lane 7) with an apparent molecular mass consistent with Hsp90 (approximately 82 kDa) and because of its established roles in fungal stress adaptation, morphogenesis, and antifungal drug tolerance. Hsp90 showed 82.7% sequence coverage in this gel band; this value is reported as an identification metric and was not used to rank proteins or infer preferential capture. Hsp90 was subsequently evaluated using independent experiments with purified recombinant protein.

Hsp90 is a particularly promising target for treating *C. albicans* infections due to its role in stress adaptation [31,69] and morphogenesis [70]. What is especially significant, Hsp90 stabilizes antifungal resistance mechanisms, and there are inhibitors of Hsp90 described in literature, which show antifungal potential, e.g., Radicicol [71]. In contrast, other proteins present in the bands cut from the gel, like ribosomal subunits or metabolic enzymes lack direct impact on pathogenicity and drug resistance.

### 3.3. Confirmation of the interaction of Hsp90 with [CoSAN]^−^ and [CoSAN-I]^−^

MALDI TOF analysis revealed that [CoSAN-I]^−^ immobilized on the chromatographic beads can bind many proteins, and Hsp90 is among them. In order to exclude the possibility of indirect binding of Hsp90 by the resin, we repeated the previous experiment using a recombinant non-tagged protein. In this study, we used resin with both immobilized [CoSAN]^−^ and [CoSAN-I]^−^. Instead of the *C. albicans* Hsp90, we used the equivalent from *Saccharomyces cerevisiae* (P02829, UniProt). Alignment of their amino acid sequences showed 81.52% identity and 92% similarity (UniProt, data not shown). Pure yeast Hsp90 was mixed with sole resin (control) as well as with [CoSAN]^−^- and [CoSAN-I]^−^-resin. Next the resins were washed with PBS buffers containing 150,300 and 500 mM NaCl. Finally, Hsp90 was eluted using an SDS-PAGE sample buffer, and protein fractions were subjected to SDS-PAGE. We observed that in the case of the two resins tested, the great majority of the pool of Hsp90 was bound to the beads (lanes 2, gels B and C, Figure 2), while sole resin bonded only a negligible portion of the target protein (lane 2, gel A, Figure 2). The result indicates that immobilized metallacarborane molecules significantly enhanced the resin–Hsp90 interaction.

**Figure 2.**
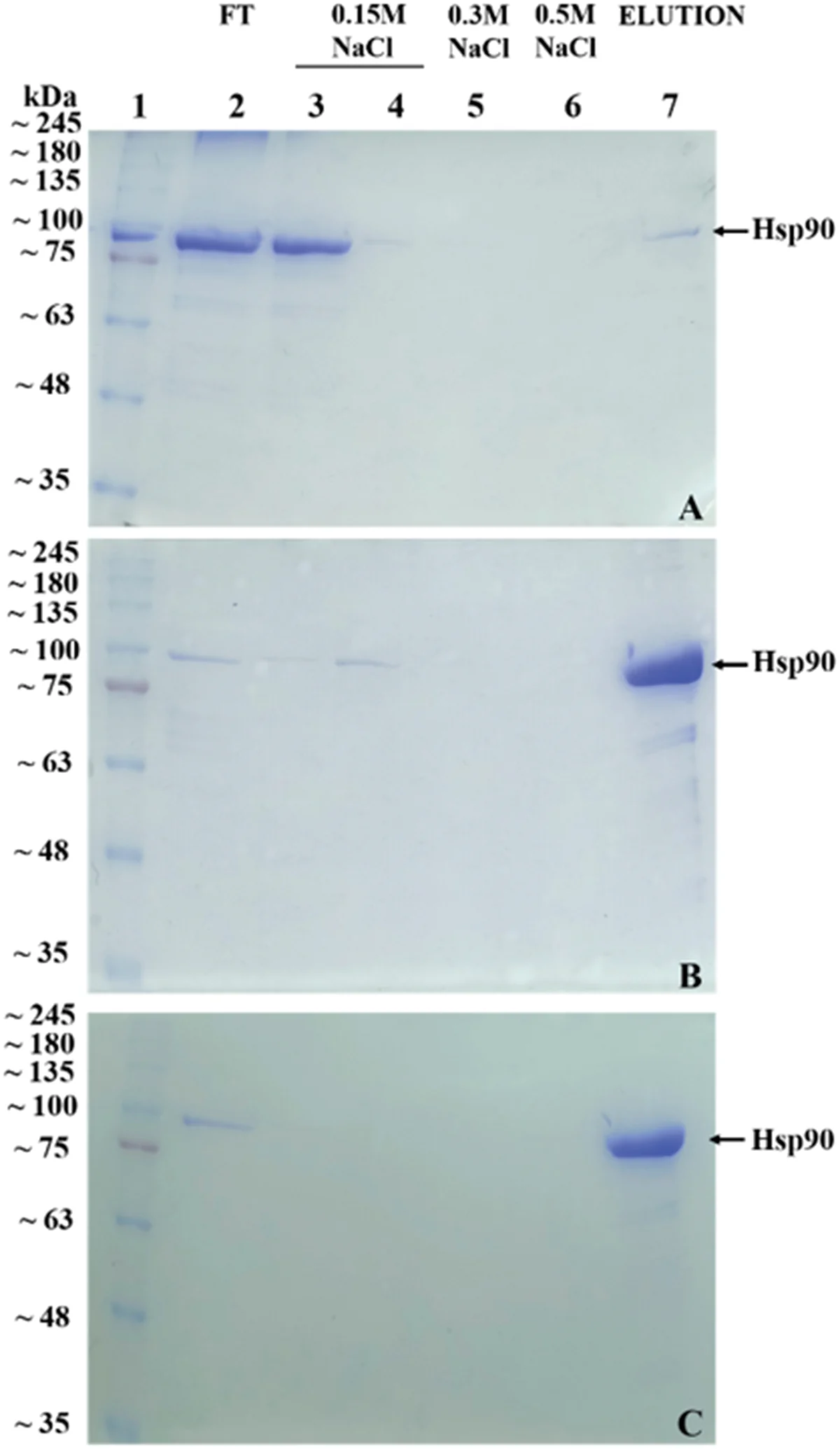
SDS-PAGE representation of the interaction of Hsp90 with the control (A), [CoSAN-I]- (B), and [CoSAN]- (C) resin. 1 – Perfect TM Tricolor Protein Ladder (EURx), 2 – fraction not bound to the resin (FT - flow-through), 3 – supernatant from the resin after first 0.15 M NaCl wash, 4 – supernatant from the resin after fifth 0.15 M NaCl wash, 5 – supernatant from the resin after 0.3 M NaCl wash, 6 – supernatant from the resin after 0.5 M NaCl wash, 7 – fraction eluted from the resin.

### 3.4. Surface plasmon resonance (SPR) interaction analysis of Hsp90 and metallacarboranes

We employed the SPR technique to measure interactions of [CoSAN]^−^, [CoSAN-I]^−^, and [CoSAN-I]^−^ derivatives (**1** and **2**) with Hsp90. Additional metallacarboranes derivatives were included, as **1** and **2** appeared to show the highest antifungal activity when previously tested against *C. albicans* strains [24]. Hsp90 was immobilized on the surface of a CM5 chip, whereas the compounds were injected over the immobilized Hsp90. An Hsp90 inhibitor, radicicol, was used as a reference. Radicicol bound to Hsp90 with the expected characteristics of a high-affinity, site-specific inhibitor (Figure 3(A,B)). The binding followed a simple 1:1 interaction model, with rapid association (association rate constant k_a_ = 8.15 ∙ 10^5^ M^−1^s^−1^), slow dissociation (dissociation rate constant k_d_ = 0.002937 s^−1^), and a low response amplitude. We calculated a dissociation constant (K_D_) to be 3.6 nM, consistent with reported literature values [72]. Additionally, we observed an initial drop in the response signal, probably due to buffer mismatch between the sample and the running buffer, which lowered the absolute values of the response upon binding to Hsp90 to 2–3 RU at the highest concentration (0.5 µM).

**Figure 3.**
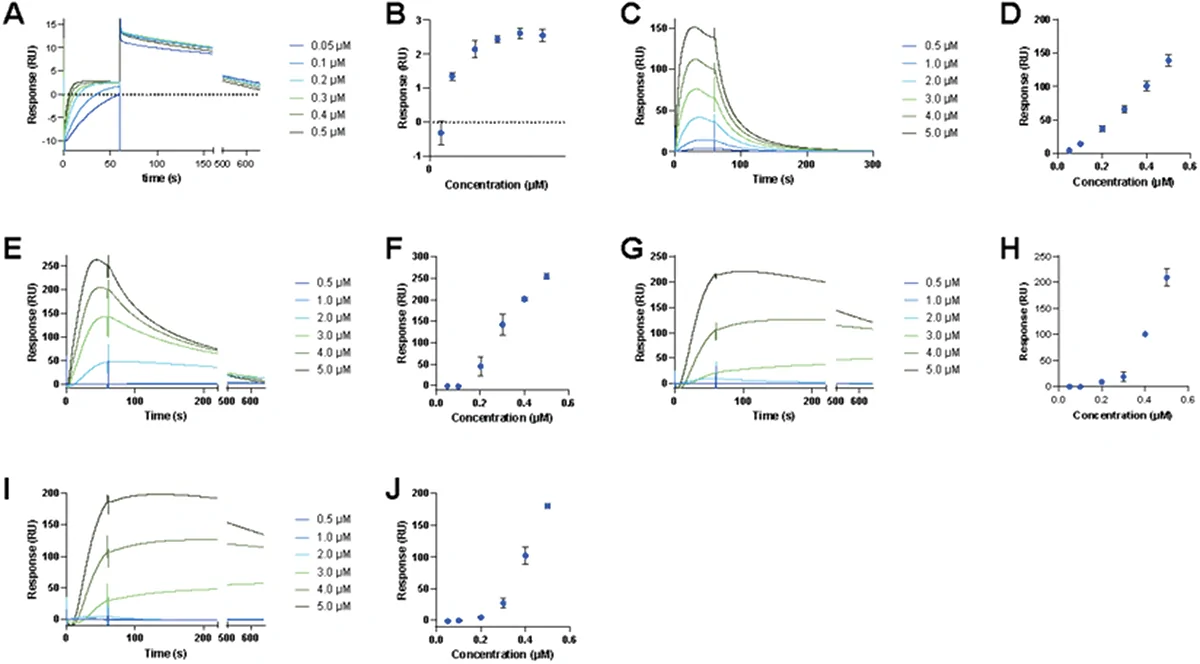
SPR sensograms showing the association and dissociation phase of complexes of Hsp90 and radicicol (A), [CoSAN]^−^ (C), [CoSAN-I]^−^ (E), 1 (G), and 2 (I). Plots of response versus concentration for radicicol (B), [CoSAN]^−^ (D), [CoSAN-I]^−^ (F), 1 (H), and 2 (J).

Metallacarboranes [CoSAN]^−^, [CoSAN-I]^−^, and derivatives **1** and **2** produced strong responses, far exceeding the theoretical R_max_ (observed responses were in the range of 150–270 RU versus expected in the range of 31–52 RU; Figure 3(C-J)). Additionally, the measured sensorgrams could not be fitted using a simple 1:1 model, suggesting heterogeneous binding with Hsp90. The sensorgrams of [CoSAN] showed a rapid increase in signal even at 0.5 µM (Figure 3(C,D)). Interestingly, during the same injection, the signal peaked and then declined before the end of the injection, followed by a rapid return to baseline during dissociation. Responses exceeding theoretical R_max_ and rapid dissociation suggest many weak and heterogeneous interactions between [CoSAN]^−^ and Hsp90.

[CoSAN-I]^−^ displayed a clear concentration threshold (Figure 3(E,F)). At concentrations below 1 µM, little or no binding was detected. Above 2 µM, signals increased sharply, reaching 250 RU at 5 µM (theoretical R_max_ ~43). Association was slightly slower than for [CoSAN]^−^, and dissociation was markedly slower. Derivatives **1** and **2** showed similar binding behavior (Figure 3(G–J)). At ≤1 µM, responses were negligible. At 2 µM, signals reached only ~10 RU during the association phase and returned quickly to baseline during the dissociation phase. However, at ≥3 µM, the kinetics changed qualitatively: the association became slow and non-saturating, with no clear plateau, and dissociation was extremely slow. Responses exceeding the theoretical R_max_ and slow dissociations suggest interaction with Hsp90 in a multivalent manner [73]. Additionally, the observed threshold effect indicates self-association in solution and subsequent interaction of Hsp90 with the aggregates. Alternatively, complexes of [CoSAN-I]^−^ or its derivatives and Hsp90 induce further aggregation of the compounds on the biosensor, resulting in heterogeneous binding [74].

The comparison between radicicol and metallacarboranes highlights distinct binding modes. Radicicol binds specifically to the ATP-binding pocket with high affinity and well-defined kinetics. In contrast, [CoSAN]^−^, [CoSAN-I]^−^, and derivatives **1** and **2** do not follow a 1:1 binding model. Their responses exceed theoretical R_max_ values and display concentration-dependent threshold effects, indicating that binding is driven by multivalency rather than a single high-affinity site.

### 3.5. Influence of metallacarborane derivatives on Hsp90 activity

Since the tested metallacarboranes physically interact with Hsp90, we assessed their influence on the catalytic activity of Hsp90. To assign the background activity of the Hsp90-catalyzed reaction, the well-known competitive inhibitor of the chaperone protein, Radicicol (Rad) at the concentration of 2 µM was added alone or in combination with [CoSAN]^−^, [CoSAN-I]^−^ and **1**. In the established reaction conditions, sole Rad inhibited the ATPase activity up to about 16%. According to Figure 4, the tested compounds were able to varying degrees overcome the effect of Rad on Hsp90. [CoSAN]^−^, [CoSAN-I]^−^ and **1** restored the ATPase activity to 61%, 65% and 72% at the concentrations of 200, 75, and 75 µM, respectively. It is worth mentioning that the modulatory potential of the compounds toward Hsp90 correlates with their antifungal potential against *C. albicans* expressed in MIC values of 120, 15 and 3.7 µM, respectively [24]. When the compounds were tested in the absence of Rad, they showed activating activity against Hsp90. The enzyme activity was maximally increased by 91%, 107%, and 82% in the presence of [CoSAN]^−^, [CoSAN-I]^−^, and **1** at the concentrations of 75, 50, and 50 µM, respectively. Higher concentrations of the metallacarborane derivatives resulted in down-regulation of Hsp90 activity. A similar behavior was observed for a different activator of the ATPase of Hsp90 identified previously [75].

**Figure 4.**
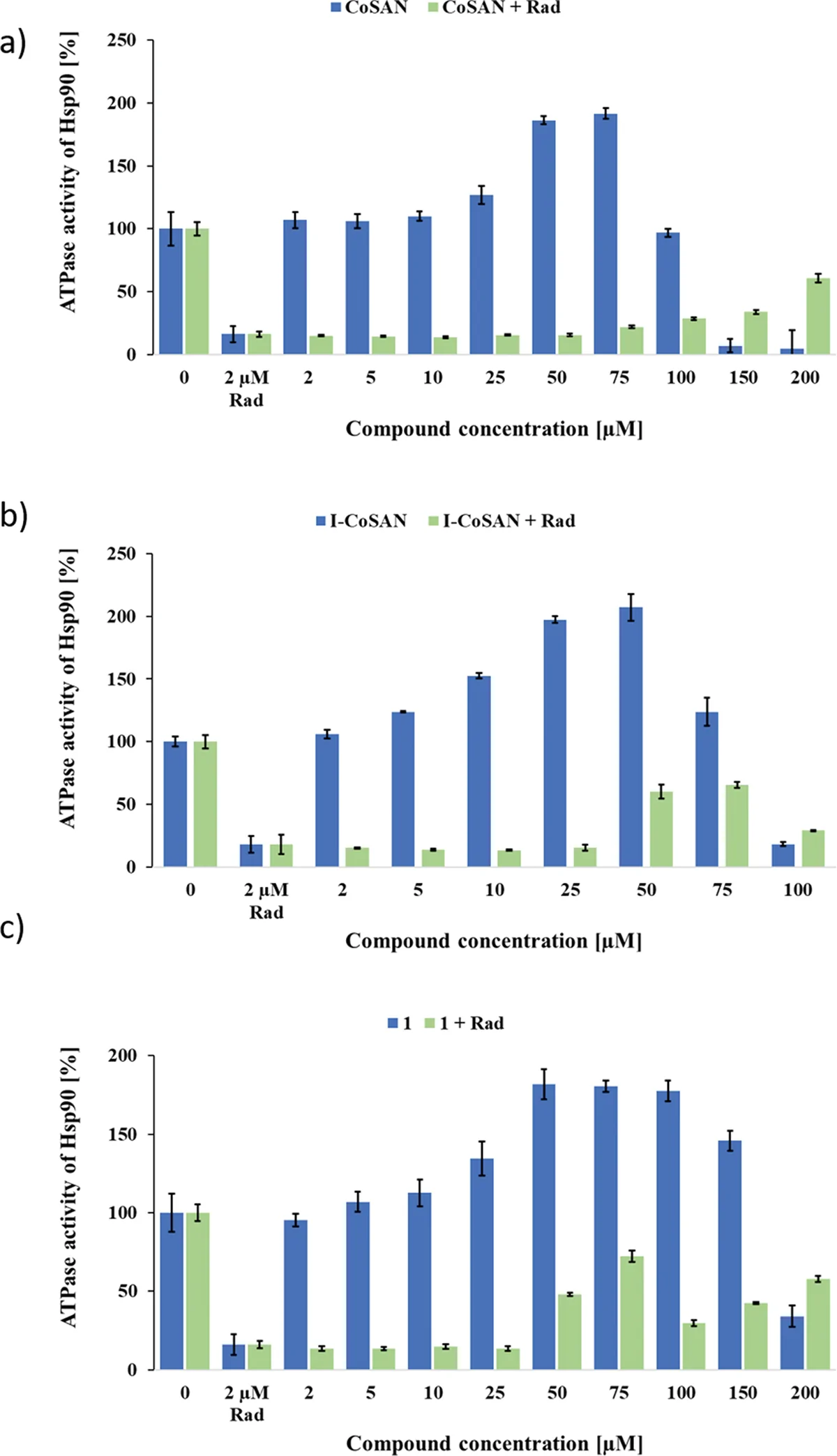
Effect of the metallacarborane derivatives [CoSAN-I]^−^, [CoSAN]^−^, and **1** on Hsp90 activity. Radicicol (Rad) was used as a control Hsp90 inhibitor.

Several limitations of the present study warrant explicit acknowledgment. First, the concentration ranges at which antifungal activity (MIC: 3.7–120 µM), direct Hsp90 binding (SPR threshold: 1–3 µM), and ATPase modulation (maximal effect at 50–200 µM) are observed do not fully overlap. This discrepancy may reflect the complexity of the Hsp90 system in a cellular context, where the abundance of co-chaperones, client proteins, and the crowded intracellular environment collectively modulate effective compound concentrations and binding equilibria. It is therefore possible that Hsp90 modulation represents one of several contributing mechanisms of antifungal action, rather than the sole determinant of cellular potency. Second, while the reversibility of the SPR signal at high compound concentrations argues against irreversible protein unfolding, ligand-induced Hsp90 aggregation at elevated concentrations cannot be entirely excluded on the basis of the present data; dynamic light scattering (DLS) measurements would provide additional structural evidence and are envisaged as part of future studies. Third, the mechanistic hypotheses proposed to explain the biphasic dose-response – allosteric N-terminal closure acceleration at low concentrations and orthosteric competition at high concentrations – remain inferential in the absence of direct structural data. Future studies employing cellular target engagement assays (e.g., thermal proteome profiling in intact *C. albicans* cells) and NMR-based binding site mapping will be required to fully resolve the molecular basis of metallacarborane–Hsp90 interactions and their relationship to antifungal efficacy.

### 3.6. What drives biphasic Hsp90 modulation? Mechanistic hypotheses

The observed biphasic behavior of the ligand, which at low concentrations accelerates and at high concentrations inhibits HSP90 protein activity, prompted us to investigate possible reasons for this phenomenon. As indicated above, Zierer et al. demonstrated similar behavior for another ATPase activator, Hsp90 [75]. The authors observed that at low concentrations, the ligand binds to an allosteric site near the NTD (N-terminal domain)/NTD–MD (middle domain), leading to an acceleration of the steps preceding the formation of the closed N-terminal state and its stabilization. In other words, this facilitates faster N-terminal closure, resulting in a more rapid formation of the hydrolyzable conformation. As ligand concentration increases, the N-terminal closure signal disappears and oligomerization occurs, indicating a blockage of the productive closed state and the hydrolysis cycle. This observation is compatible with allosteric modulation, although the binding site has not been directly mapped. The authors note that the ligand participates in the modification of the orthosteric site and triggers activation even in the absence of an ATP molecule. This is not an isolated case, as in his doctoral thesis, Stiegler describes an equally interesting observation that one of the HSP90 inhibitors acts as an ATP activator of HSP90 in the absence of the cochaperone Aha1 [76]. This indicates a unique type of interaction with HSPs, the mechanism of which depends on whether Hsp90 is in complex with cochaperone or not.

Taking into account the dose-dependent biphasic activity of our ligands and literature reports, it can be concluded that one of the main mechanisms responsible for this phenomenon is the participation of the ligand in the rate-limiting step of the reaction. At low concentration, the ligand may accelerate a rate-limiting conformational step of Hsp90 - N-terminal closure - which precedes hydrolysis. At higher concentrations, nonproductive modulation of Hsp90 conformational cycling may contribute to reduced activity preventing the correct completion of the cycle. A study by Zierer et al., using the glucocorticoid receptor (GR) as a model client, indicated that coordination of the cycle time and affinity for the client is crucial for the effectiveness of the HSP90 system. Therefore, both acceleration and inhibition of the system disrupt information processing by the client [75]. Similarly, the studies by Koulov et al. proposed that Aha1 modulates the coupling between the Hsp90 ATPase cycle and the fate of the client by regulating the client’s dwell time within the chaperone complex, thereby shifting the balance between efficient folding and degradation dependent on folding quality [77]. Therefore, the duration of interaction may be a key factor determining the correct cycle and, therefore, activity. Furthermore, it was hypothesized that Aha1 may also influence cell maturation by modulating the dwell time of Hsp90 clients [78]. It has also been shown that some Hsp90 clients influence the rate of Hsp90 activity, which is attributed to conformational stabilization in ATPase-competent or incompetent states, respectively. This indicates that clients modulate their residence time in Hsp90 [38,78–80]. McLaughlin et al. suggested that tight regulation of the Hsp90 ATPase activity allows the client protein to remain bound to Hsp90 for a sufficient period of time for activation to occur [81]. This suggests that, if not properly regulated, the time required for activation may also be suboptimal. This mechanism can be considered one likely explanation for the biphasic dose-dependent effect of our ligands.

Zierer et al. found that titration with a compound exhibiting biphasic activity resulted in poor solubility and a tendency to aggregate, which is why NMR mapping of the binding site was not possible [75]. Such analysis was performed for another activator, which indicated that it binds near the α2 helix and subsequently induces conformational changes in the ATP binding site [75]. Given the proximity of allosteric and orthosteric sites and the unique nature of metallacarboranes (described below), it is reasonable to hypothesize that they may bind to both sites, which is concentration dependent. At low concentrations, the allosteric site is occupied, which leads to the acceleration of kinase activity; when the concentration increases after exceeding the critical point characteristic for a given compound, metallacarboranes can not only stabilize the closed conformation, but also direct competition at the orthosteric site cannot be excluded. In this case, metallacarboranes compete with the ATP molecule for the binding site. To attempt to further elucidate the mechanisms underlying this phenomenon, a structural analysis using quantum chemical methods was performed in the next chapter. Because it is impossible to pinpoint the exact allosteric site responsible for the acceleration of Hsp90 activity (Zierer et al. only identified a broad region where this site may be located [75]), molecular docking was performed only to the orthosteric site.

Overall, the mechanistic interpretation presented above should be regarded as a working hypothesis rather than a direct conclusion from the current experiments. The observed biphasic modulation of Hsp90 ATPase activity is consistent with concentration-dependent effects on the Hsp90 conformational cycle; however, the relative contributions of allosteric modulation, potential orthosteric competition, multivalent binding, and compound self-association or ligand-induced aggregation cannot be resolved by the present data. Direct binding-site mapping and complementary biophysical studies will be required to distinguish among these possibilities.

### 3.7. Metallacarboranes as unique ligands – *in silico* investigation

Based on the above-mentioned mechanism of activity, we focused on analyzing data concerning the unusual properties of metallacarboranes. Our attention was initially drawn to the work of Kaplánek et al. who found that in the case of nitric oxide synthase, boron cluster compounds can act as activators or inhibitors, and this is dependent on peripheral substitution of the boron cluster compound [82]. In a later addition, Kožíšek et al. indicate that for the simplest metallacarborane derivative, [CoSAN]^−^, two different binding modes are observed in the HIV protease at identical binding sites due to a degree of freedom in its binding position. Furthermore, the metallacarborane cage can adjust its position in response to changes in the binding pocket [83]. This means that both effects are caused by the unusual structure of metallacarboranes.

To elucidate the electronic landscape of the ligand, we conducted a specific analysis using Density Functional Theory (DFT). Following geometry optimization, the frontier molecular orbitals (FMOs) – specifically the Highest Occupied Molecular Orbital (HOMO) and the Lowest Unoccupied Molecular Orbital (LUMO) – were examined to evaluate the molecule’s global chemical reactivity. As the HOMO and LUMO energies govern electron-donating and electron-accepting capabilities, respectively, their topological characterization provides critical insight into the potential interactions with a potential molecular target [84,85]. Figure S3 shows visualizations of the HOMO and LUMO orbitals for a) [CoSAN]^−^, b) [I-CoSAN]^−^, and c) 1, respectively.

In the case of [CoSAN]^−^, the HOMO orbital localizes unevenly, encompassing both dicarbolides. A distinctly strong localization occurs near the cobalt ion. However, the LUMO orbital is uniformly covered throughout the molecule. In the case of [CoSAN-I]^−^, the HOMO orbital organizes primarily on the iodine-substituted carbolide, specifically on the iodine atom, its surroundings, and the cobalt ion. In the case of the second carbolide, the orbital is located on the opposite side of the iodine atom. The LUMO orbital, on the other hand, uniformly covers the entire molecule, excluding the iodine atom.

In the case of **1**, the HOMO orbital is concentrated primarily on the cyclohexanoxy-substituted carbolide cage, particularly near the ether oxygen atom. Similarly, in the cyclohexane ring, it is half-occupied by this orbital, particularly at the linker attachment site. Significant intensity of this orbital also occurs near Co^3+^ and the iodine atom. In this case, the dicarbolide cage is not uniformly covered by the HOMO orbital but is shifted toward the iodine atom, similarly to [CoSAN-I]^−^. The LUMO orbital is evenly distributed between both carbolides, without loss of orbital localization on the iodine atom or the cyclohexane ring. There is minor localization on the ether oxygen atom.

Table 2 presents the HOMO, LUMO, and LUMO-HOMO gap energy values for given compounds. The GAP energy determines the reactivity of the molecule and is inversely proportional to the energy gap value. For high values, the molecule is characterized by high stability [85,86].

**Table 2.** HOMO, LUMO, and LUMO–HOMO gap energy values for given compounds.

	[CoSAN]−	[CoSAN-I]−	1
HOMO [eV]	−9.203	−8.925	−8.618
LUMO [eV]	−0.132	−0.324	−0.207
Energy Gap [eV]	9.071	8.601	8.411

The determined LUMO-HOMO energy gap values for the studied compounds are in the range of 8.6–9.0 eV. Comparative analysis with data from the QM9 database for organic molecules [https://doi.org/10.1038/sdata.2014.22] demonstrates that the studied derivatives are characterized by above-average stability, significantly exceeding the typical values for drugs (4–6 eV) [87]. According to the HSAB theory, the ‘hard–hard’ interaction mechanism dominates, based primarily on electrostatic and dispersive components, without significant contribution from charge transfer effects [88]. A detailed structure–activity relationship (SAR) analysis reveals a nonlinear correlation between the calculated energy gap and biological activity. [CoSAN-I]^−^ exhibits the highest activity (8.601 eV), which can be attributed to the synergy of the stable dicarbolide cage and the presence of the iodine atom. The moderate activity of [CoSAN]^−^ (9.071 eV) results from the dominance of pure electrostatic interactions. The lower activity for **1** (despite the lowest gap of 8.411 eV) demonstrates the decisive role of steric factors – the introduction of the bulky cyclohexyl group likely generates steric clash, which, according to QSAR (quantitative structure–activity relationship) principles, prevents effective binding despite the favorable electronic potential [89]. The next stage of our research devoted to structural analysis was the evaluation of the molecular electrostatic potential (MEP) distribution. MEP describes the charge distribution in a molecule and allows for the quantitative identification of electrophilic and nucleophilic regions, which are crucial for the formation of non-covalent interactions with molecular targets [90]. Figure S3 shows the molecular electrostatic potential generated based on the calculated electron density.

Electrostatic potential maps reflect the charge density distribution in the tested anionic metal carborane clusters. For [CoSAN]^−^, the potential is distributed evenly, with a dominance of a slightly negative charge delocalized on the cage surface (Figure S3(d)). On one side there is a local accumulation of a sphere with a charge close to zero or even slightly positive, and on the other side there is a local small accumulation of a more negative sphere. For [CoSAN-I]^−^, a local enhancement of the negative potential is visible on the iodine atom, and on the opposite side - of the opposite charge. Naturally, a slightly negative charge dominates (Figure S3(e)). The most visible differentiation, different from [CoSAN]^−^ and [CoSAN-I]^−^, occurs in derivative **1**, where the iodine atom, corresponding to slightly positive potential values, while the negative charge accumulates within the cyclohexyl substituent (Figure S3(f)). The slightly negative charge extends from the opposite side carbolide cage with respect to the iodine atom along with the entire other opposite carbolide. The distinctly different nature of the potential distribution for **1** may explain its significantly lower activity relative to the other two derivatives.

Following our confirmation of boron cluster interaction with Hsp90, and the impact of these molecules on ATPase activity, it becomes necessary to elucidate the mode of interaction and the formation of the ligand-protein complex. To more comprehensively understand the activity of our compounds, molecular docking analysis was performed to assess their potential ligand–target interactions. As mentioned earlier, molecular docking analysis only refers to interactions with the orthosteric site.

There are several papers in the literature devoted to molecular docking of carborane [91–94] and metallacarborane derivatives [94,95]. In this paper, we present a new, simplified approach, which was validated using redocking and cross-validation method (for details, please refer to the methodology section).

Figure 5(a) illustrates the superposition of the analyzed derivatives within the binding pocket, highlighting their spatial arrangement relative to amino acid residues. Subsequently, Figure 5(b) depicts the conformational impact of structural modifications on the ligand orientation within the active site. Figure 5(c-e) shows the docked compounds with nearby amino acid residues with which non-covalent interactions can form. Essentially, all three ligands occupy the same position in the orthosteric binding site of HSP90. Even the introduction of substituents such as an iodine atom ([CoSAN-I]^−^) or a cyclohexaneoxy substituent (**1**) does not change the position of the ligand relative to the unsubstituted one ([CoSAN]^−^). This effect may be explained by the highly hydrophobic nature of metallacarboranes [16], which causes them to also locate in the hydrophobic part of the binding chamber [28]. Therefore, their interactions with the molecular target also have the same specificity. This was confirmed by the electron density analyses described above.

**Figure 5.**
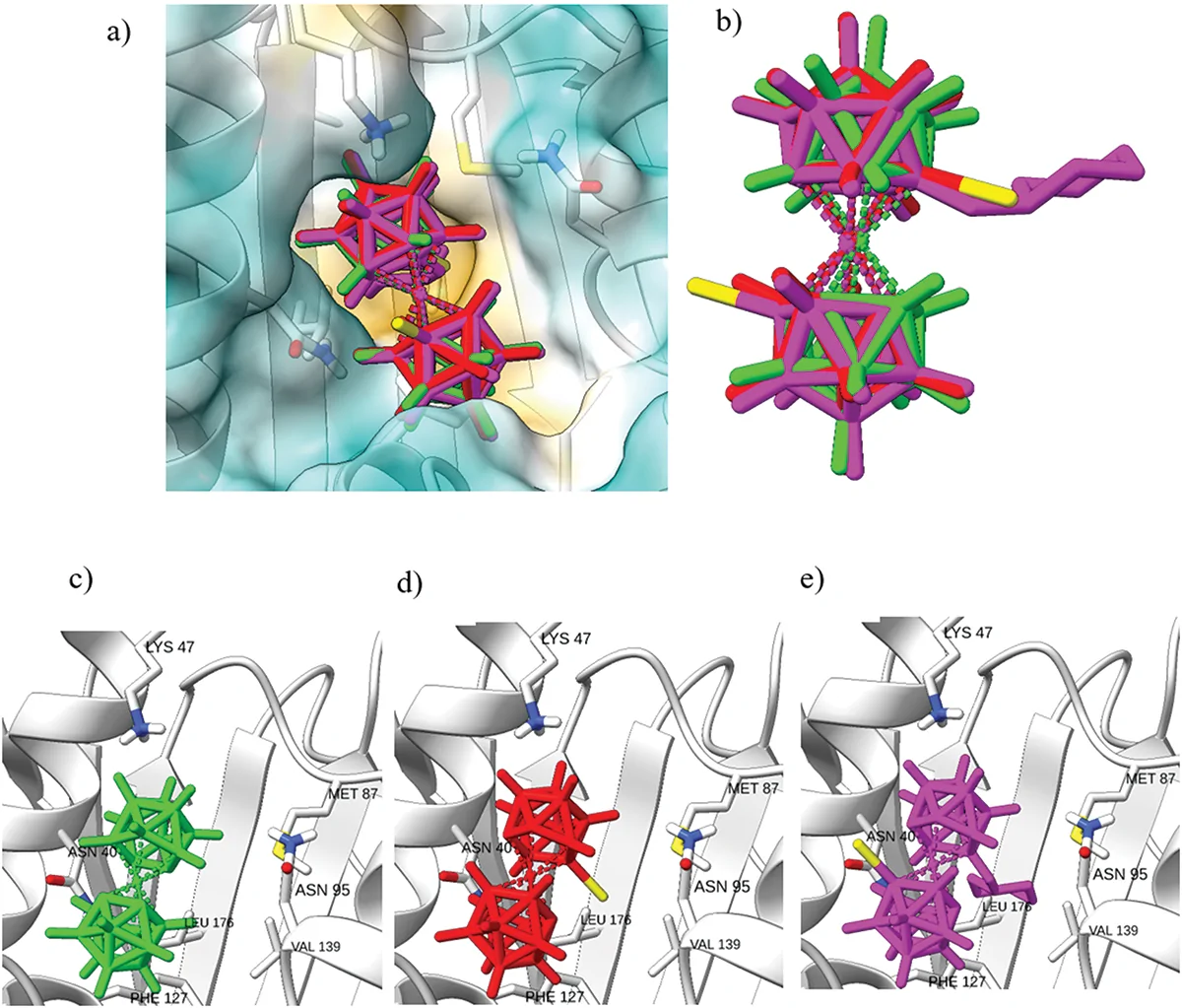
(a) Superimposed of the docked compounds ([CoSAN]^−^ - lime, [CoSAN-I]^−^ red, and 1-magenta). The iodine atom is shown in yellow. (b) Arrangement of the compounds in the active site (the active site has been removed for clarity). Only nonpolar hydrogen atoms are presented; (c) [CoSAN]^−^, (d) [CoSAN-I]^−^, and (e) **1** together with close amino acid residues. Only nonpolar hydrogen atoms are presented.

Differences in the arrangement of the atoms of [CoSAN-I]^−^ and **1** in the binding cavity were observed. The iodine atom is directed into the cavity toward VAL139, while in case **1** it is directed toward the exit of the binding cavity, respectively. Interestingly, in case **1**, the dicarbolide cage is also inverted relative to the [CoSAN-I]^−^ arrangement. The cyclohexanoxy substituent is located in the hydrophobic part of the cavity and creates much stronger interactions than the less hydrophobic iodine atom, which may explain the different arrangement in the binding site compared to [CoSAN-I]^−^. Additionally, the presence of the ether oxygen atom may be crucial for the fit of the cyclohexyl ring in the binding cavity; similar observations were made for the ether oxygen atom of the phenoxy group [96]. The strength of interactions with the molecular target increases in the following order: [CoSAN]^−^ (−0.73 kcal/mol) < [CoSAN-I]^−^ (−0.94 kcal/mol) < **1** (−2.10 kcal/mol). It should be remembered that these are only predicted interaction energies. Regarding the amino acid residues with which the tested compounds form potential hydrophobic interactions, the same trends are observed for all compounds. However, one of the dicarbolides can only form interactions with PHE127 and, to a lesser extent, ASN40, while the other can only form interactions with ASN40, LYS47, MET87, and ASN95. Moreover, for the cyclohexane ring of compound **1** it is possible to form interactions with VAL139 and LEU176. The location in closer proximity to the amino acid residues that will allow for the formation of interactions is justified by the ESP potential described above. Among the possible hydrophobic interactions mentioned above, the main expected interactions are van der Waals interactions, BH–π and CH–π interactions with aromatic amino acids, and contacts with aliphatic amino acids [16,97]. *In silico* analysis confirms the unusual behavior of metallacarboranes and provides insight into their unique parameters that may account for the binding at two sites depending on concentration.

## 4. Conclusions

The work presented herein marks a significant step toward understanding the molecular activity of metallacarboranes in antifungal therapy. By selecting derivatives that are active against both reference and drug-resistant *C. albicans* isolates and employing affinity capture with immobilized [COSAN]^−^ and [CoSAN-I]^−^, we have successfully identified several protein targets. Among these, the Hsp90 emerges as a critical and novel target, given its central role in fungal virulence, stress response, and resistance pathways. Data from affinity chromatography, surface plasmon resonance, and ATPase inhibition, supported by *in silico* calculations, underscore the high potential of [CoSAN]^−^ derivatives as modulators of Hsp90 function. The findings not only expand the repertoire of boron-cluster engagements with biological macromolecules, but also open a rational design pathway for boron-cluster-based therapeutics with enhanced selectivity and efficacy.

## Supplementary Material

Supplementary Material.docx

## Data Availability

SPR and MALDI-TOF data were deposited at https://osf.io/7qyka/overview?view_only=3cc451ae4db84c85851810ab1463f4cf. Other data is available on request.

## Article highlights

- Candidiasis caused by *Candida albicans* represents a growing global health threat, with rising resistance to all major antifungal drug classes. Novel therapeutic strategies targeting unexplored molecular mechanisms are urgently needed.
- Using [CoSAN-I]^−^-functionalized affinity chromatography resin, we performed the first proteome-wide screen for metallacarborane-interacting proteins in *C. albicans*. MALDI-TOF analysis identified 72 proteins, of which 14 have established roles in antifungal drug action, fungal virulence, or stress adaptation.
- The molecular chaperone Hsp90 – a master regulator of *C. albicans* morphogenesis, biofilm formation, and antifungal resistance – was selected as a candidate from the CoSAN-I affinity-capture screen and its direct interaction with metallacarboranes was subsequently validated using purified protein and SPR.
- SPR revealed multivalent, heterogeneous binding of all metallacarborane derivatives to Hsp90 (responses of 150–270 RU vs. theoretical R~max~ of 31–52 RU), contrasting with the classical 1:1 binding of reference inhibitor radicicol (K~D~ = 3.6 nM).
- All compounds modulated Hsp90 ATPase activity in a biphasic, dose-dependent manner (maximal activation 82–107% at 50–75 µM), with modulatory potency correlating with antifungal activity rank order. DFT calculations and molecular docking support a dual allosteric/orthosteric binding mechanism.
- This study establishes Hsp90 as a direct molecular interactor of antifungal metallacarboranes, providing the first mechanistic framework for rational design of boron-cluster-based antifungal therapeutics.

## Writing assistance

No writing assistance was utilized in the production of this manuscript.
