# Supplementary material for: Antifungal metallacarboranes modulate Hsp90 activity
Source: Future Med Chem. 2026 Sep 21;18(19):2713–29. doi: 10.1080/17568919.2026.2732258 (PMC13641588; doi:10.1080/17568919.2026.2732258)
Supplement: Supplementary file 1 [file IFMC_A_2732258_SM6178.docx]

Antifungal Metallacarboranes Modulate Hsp90 Activity

**Table S1**. The list of proteins present in the analyzed bands with a threshold of 50% coverage used.

| Protein Name | Maximum Sequence Coverage in a Single Band (%) | UniProtKB Entry | Cellular Localization | Main Funktion | Antifungal Target (Yes/No) |
| --- | --- | --- | --- | --- | --- |
| 10 kDa heat shock protein, mitochondrial | 69,8 | A0A1D8PFU8 | Mitochondrion (matrix) | Small mitochondrial heat shock chaperone preventing aggregation of unfolded proteins | No |
| 26S protease regulatory subunit 8 | 55,1 | C4YKF8 | Nucleus, Cytoplasm | ATP-dependent regulatory subunit of the 26S proteasome involved in ubiquitin-mediated protein degradation | No |
| 26S proteasome regulatory subunit RPN8 | 57,6 | C4YR89 | Nucleus, Cytoplasm | Lid subunit of the 19S proteasome regulatory particle required for substrate recognition and deubiquitination | No |
| 3-hydroxy-3-methylglutaryl coenzyme A synthase | 75,8 | A0A1D8PTW6 | Cytoplasm | Catalyzes HMG-CoA synthesis in the mevalonate/isoprenoid biosynthesis pathway | Yes (Essential metabolic protein (ergosterol/isoprenoid biosynthesis pathway)) |
| 4-amino-5-hydroxymethyl-2-methylpyrimidine phosphate synthase | 60,2 | Q5A3Y5 | Cytoplasm | Catalyzes pyrimidine moiety synthesis during thiamine (vitamin B1) biosynthesis | No |
| 40S ribosomal protein S0 | 71,3 | O42817 | Cytoplasm | Structural component of the 40S ribosomal subunit involved in translation | No |
| 40S ribosomal protein S1 | 52,3 | A0A367XRX6 | Cytoplasm | Structural constituent of the 40S ribosomal subunit | No |
| 40S ribosomal protein S12 | 68,5 | Q5ADQ6 | Cytoplasm | Structural constituent of the 40S ribosome; protein synthesis | No |
| 40S ribosomal protein S15 | 71,1 | C4YQM1 | Cytoplasm | Structural constituent of the 40S ribosome | No |
| 40S ribosomal protein S17-B | 78,8 | C4YCW2 | Cytoplasm | Structural constituent of the 40S ribosome | No |
| 40S ribosomal protein S18 | 67,6 | C4YTG2 | Cytoplasm | Structural constituent of the 40S ribosome | No |
| 40S ribosomal protein S19-B | 68,1 | M3K6B6 | Cytoplasm | Structural constituent of the 40S ribosome | No |
| 40S ribosomal protein S24 | 58,5 | Q5A7K0 | Cytoplasm | Structural constituent of the 40S ribosome | No |
| 40S ribosomal protein S3 | 66,9 | C4YLU5 | Cytoplasm | Structural constituent of the 40S ribosome; mRNA decoding | No |
| 40S ribosomal protein S7 | 58,1 | Q5AJ93 | Cytoplasm | Structural constituent of the 40S ribosome | No |
| 40S ribosomal protein S8 | 52,4 | Q59T44 | Cytoplasm | Structural constituent of the 40S ribosome | No |
| 60S acidic ribosomal protein P0 | 54,8 | G1UA71 | Cytoplasm | Acidic ribosomal stalk protein required for translation elongation | No |
| 60S acidic ribosomal protein P1-alpha | 63,4 | C4YEX4 | Cytoplasm | Acidic ribosomal stalk protein involved in recruitment of translation factors | No |
| 60S acidic ribosomal protein P1-B | 68,5 | G1UAZ3 | Cytoplasm | Acidic ribosomal stalk protein involved in translation elongation | No |
| 60S ribosomal protein L13 | 58,4 | C4YEX3 | Cytoplasm | Structural constituent of the 60S ribosomal subunit | No |
| 60S ribosomal protein L25 | 58,5 | C4YSV1 | Cytoplasm | Structural constituent of the 60S ribosomal subunit | No |
| 60S ribosomal protein L26-B | 66,9 | C4YF39 | Cytoplasm | Structural constituent of the 60S ribosomal subunit | No |
| 60S ribosomal protein L28 | 72,5 | C4YLC1 | Cytoplasm | Structural constituent of the 60S ribosomal subunit | No |
| 60S ribosomal protein L30 | 74,8 | C4YGY4 | Cytoplasm | Structural constituent of the 60S ribosomal subunit | No |
| 60S ribosomal protein L4-B | 63,9 | C4YFR7 | Cytoplasm | Structural constituent of the 60S ribosomal subunit | No |
| 60S ribosomal protein L6 | 51,7 | Q9P834 | Cytoplasm | Structural constituent of the 60S ribosomal subunit | No |
| Acetolactate synthase small subunit, mitochondrial | 59,4 | C4YHU4 | Mitochondrion (matrix) | Catalyzes the first step of branched-chain amino acid biosynthesis | No |
| Acetyl-CoA C-acetyltransferase | 57,7 | A0A1D8PH52 | Cytoplasm, Peroxisome | Catalyzes condensation of acetyl-CoA molecules during lipid and isoprenoid metabolism | No |
| Actin | 71,2 | C4YFV6 | Cytoplasm, Cytoskeleton | Major actin cytoskeleton component involved in cell polarity, morphogenesis and cytokinesis | No |
| Actin-related protein 2/3 complex subunit 5 | 63,9 | Q59RN2 | Cytoplasm, cortical actin cytoskeleton | Component of the Arp2/3 complex regulating actin filament nucleation and branching | No |
| Actin-related protein 3 | 50,4 | Q59Z11 | Cytoplasm, cortical actin cytoskeleton | Core subunit of the Arp2/3 complex involved in actin polymerization | No |
| Alcohol dehydrogenase | 80 | Q27J13 | Cytoplasm | Catalyzes reversible oxidation of alcohols and aldehydes during carbon metabolism | No |
| Alcohol dehydrogenase (Fragment) | 73,5 | M1T132 | Cytoplasm | Alcohol dehydrogenase involved in alcohol metabolism (fragment) | No |
| Alcohol dehydrogenase I | 70,3 | C4YS13 | Cytoplasm | NAD-dependent alcohol dehydrogenase involved in fermentative metabolism | No |
| Anthranilate phosphoribosyltransferase | 61,3 | C4YGE3 | Cytoplasm | Catalyzes anthranilate phosphoribosylation in tryptophan biosynthesis | No |
| Antisense-enhancing sequence 1 | 85,8 | A0A1D8PQ54 | Nucleus | RNA-binding protein involved in RNA processing and regulation of gene expression | No |
| Arf family GTPase | 62,4 | Q5AND9 | Cytoplasm, Golgi membranes | Small GTPase regulating vesicle trafficking and membrane transport | No |
| ATP synthase subunit beta | 78 | A0A1D8PKZ9 | Mitochondrion (inner membrane) | Catalytic β subunit of mitochondrial ATP synthase responsible for ATP production | Yes (Essential protein (energy metabolism)) |
| ATP-dependent molecular chaperone HSP82 | 82,7 | C4YTQ8 | Cytoplasm | ATP-dependent Hsp90 family molecular chaperone involved in protein folding and stress response | Yes (Experimental antifungal target; virulence factor; regulator of antifungal resistance) |
| ATP-dependent RNA helicase SUB2 | 52 | C4YI53 | Nucleus | ATP-dependent RNA helicase required for pre-mRNA splicing and mRNA export | No |
| Bifunctional cysteine synthase/O-acetylhomoserine aminocarboxypropyltransferase | 83,6 | Q59US5 | Cytoplasm | Catalyzes sulfur amino acid biosynthesis through cysteine and homocysteine metabolism | No |
| Bifunctional phosphoribosylaminoimidazolecarboxamide formyltransferase/IMP cyclohydrolase | 55,1 | Q5A6R2 | Cytoplasm | Bifunctional enzyme catalyzing late steps of de novo purine biosynthesis | No |
| Calcineurin subunit B | 50,3 | C4YS24 | Cytoplasm | Calcium-binding regulatory subunit of calcineurin phosphatase | No |
| Cam1-1p | 83,7 | A0A1D8PNN8 | Cytoplasm, bud neck | Calmodulin involved in calcium signaling, cytokinesis and polarized growth | No |
| Catabolic 3-dehydroquinase | 51,4 | C4YK27 | Cytoplasm | Catalyzes conversion of 3-dehydroquinate in aromatic amino acid biosynthesis | No |
| Chaperone ATPase | 77,9 | A0A1D8PTP9 | Cytoplasm | AAA+ ATPase chaperone involved in protein quality control | No |
| Chaperonin-containing T-complex alpha subunit | 54 | Q59QB7 | Cytoplasm | CCT/TRiC chaperonin α subunit required for folding of actin and tubulin | No |
| Chaperonin-containing T-complex subunit | 67,5 | Q59YC4 | Cytoplasm | CCT/TRiC chaperonin subunit involved in folding of cytoskeletal proteins | No |
| Chaperonin-containing T-complex subunit | 61,7 | Q5AB74 | Cytoplasm | CCT/TRiC chaperonin subunit involved in ATP-dependent protein folding | No |
| Chorismate synthase | 56,7 | A0A1D8PTK1 | Cytoplasm | Catalyzes the final step of chorismate biosynthesis in the shikimate pathway | No |
| Cip1p | 81,6 | A0A1D8PPI6 | Cytoplasm | Cell cycle regulatory protein involved in stress-induced cell cycle arrest | No |
| Coproporphyrinogen oxidase | 61 | Q59MR4 | Mitochondrion | Catalyzes oxidative decarboxylation of coproporphyrinogen III during heme biosynthesis | No |
| COX assembly mitochondrial protein | 51,9 | Q5ALU3 | Mitochondrion (inner membrane) | Assembly factor required for cytochrome c oxidase biogenesis | No |
| Cystathionine gamma-lyase | 53,9 | Q5A362 | Cytoplasm | Catalyzes conversion of cystathionine to cysteine in sulfur amino acid biosynthesis | No |
| Cytochrome c oxidase assembly factor 6 | 58,3 | C4YN47 | Mitochondrion (inner membrane) | Cytochrome c oxidase assembly factor required for respiratory complex IV biogenesis | No |
| Cytochrome c oxidase subunit VI | 67,3 | A0A1D8PH08 | Mitochondrion (inner membrane) | Structural subunit of cytochrome c oxidase (Complex IV) | No |
| D-3-phosphoglycerate dehydrogenase | 79,1 | Q5A3K7 | Cytoplasm | Catalyzes the first step of serine biosynthesis from 3-phosphoglycerate | No |
| DNA mismatch repair protein HSM3 | 54,2 | C4YSY2 | Nucleus | Component of the mismatch repair machinery involved in genome stability | No |
| DNA-directed RNA polymerase II 13.6 kDa polypeptide | 74,2 | C4YFG3 | Nucleus | RNA polymerase II subunit required for transcription of protein-coding genes | No |
| Elongation factor 1-alpha | 88,2 | C4YDJ3 | Cytoplasm | Translation elongation factor delivering aminoacyl-tRNAs to the ribosome during protein synthesis | Yes (Essential protein (protein synthesis)) |
| Elongation factor 2 | 81 | Q5A0M4 | Cytoplasm | GTP-dependent translation elongation factor mediating ribosomal translocation | Yes (Experimental antifungal target; essential protein (protein synthesis)) |
| Elongation factor Tu | 51,9 | Q5ABC3 | Mitochondrion | Mitochondrial elongation factor Tu required for mitochondrial protein translation | No |
| Enoyl reductase (ER) domain-containing protein | 51,9 | C4YGI1 | Endoplasmic reticulum | Enoyl reductase domain involved in fatty acid biosynthesis | No |
| Eukaryotic initiation factor 4A | 88,7 | C4YFC5 | Cytoplasm | ATP-dependent RNA helicase involved in translation initiation | No |
| Farnesoic acid-induced protein 2 | 66,7 | E2ITF0\| | Cytoplasm | Stress-responsive protein implicated in adaptation to environmental stress | No |
| Fructose-2,6-bisphosphatase | 51,5 | Q5A340 | Cytoplasm | Regulates fructose-2,6-bisphosphate levels and glycolytic flux | No |
| Gfo/Idh/MocA-like oxidoreductase N-terminal domain-containing protein | 87,6 | C4YSP4 | Cytoplasm | NAD(P)-dependent oxidoreductase involved in cellular redox metabolism | No |
| Glutamate decarboxylase | 68,8 | A0A1D8PF79 | Cytoplasm | Catalyzes conversion of glutamate to γ-aminobutyrate (GABA) | No |
| Glutamate decarboxylase (Fragment) | 55,8 | B2B9P3 | Cytoplasm | Glutamate decarboxylase involved in GABA biosynthesis (fragment) | No |
| Glutamate-5-semialdehyde dehydrogenase | 51,6 | Q5ADR2 | Mitochondrion | Catalyzes glutamate semialdehyde oxidation during proline biosynthesis | No |
| Glutamine synthetase | 86,6 | A0A1D8PSY1 | Cytoplasm | ATP-dependent synthesis of glutamine from glutamate and ammonia | No |
| Glyceraldehyde-3-phosphate dehydrogenase | 81,5 | Q5ADM7 | Cytoplasm | Glycolytic enzyme converting glyceraldehyde-3-phosphate to 1,3-bisphosphoglycerate | No |
| Glycerol 2-dehydrogenase (NADP(+)) | 57 | Q5ADT4 | Cytoplasm | NADP-dependent glycerol dehydrogenase involved in glycerol metabolism | No |
| Glycerol-3-phosphate dehydrogenase [NAD(+)] | 82 | Q8NKG1 | Cytoplasm, mitochondrion | Converts glycerol-3-phosphate and dihydroxyacetone phosphate in glycerol metabolism | No |
| Glycine--tRNA ligase | 54,6 | Q5A2A5 | Cytoplasm | Charges tRNAGly with glycine during protein synthesis | No |
| Gre2p | 69,3 | A0A1D8PHQ6 | Cytoplasm | NADPH-dependent reductase involved in oxidative and osmotic stress response | No |
| Gst2p | 66,7 | Q5AFB4 | Cytoplasm | Glutathione S-transferase involved in detoxification and oxidative stress defense | No |
| Heat shock protein 104 | 77,9 | C4YMN0 | Cytoplasm | AAA+ disaggregase that resolubilizes aggregated proteins after stress | No |
| Heat shock protein 60, mitochondrial | 80 | C4YM75 | Mitochondrion (matrix) | Mitochondrial chaperonin Hsp60 required for protein folding | No |
| Heat shock protein 70 | 63,9 | G9BX82 | Cytoplasm | Hsp70 family molecular chaperone involved in protein folding and stress response | No |
| Heat shock protein 70 (Fragment) | 60,4 | D2J4C7 | Cytoplasm | Hsp70 molecular chaperone (fragment) | No |
| Heat shock protein 78, mitochondrial | 55,2 | Q96UX5 | Mitochondrion | ATP-dependent mitochondrial chaperone involved in protein quality control | No |
| Heat shock protein Hsp104 | 64,5 | Q96W69 | Cytoplasm | ATP-dependent Hsp104 disaggregase involved in thermotolerance | No |
| Heat shock protein SSA1 | 78,2 | P41797 | Cytoplasm | Cytosolic Hsp70 chaperone involved in protein folding and stress adaptation | Yes (Virulence factor; stress-response protein) |
| Heat shock protein SSA2 | 77,1 | P46587 | Cytoplasm | Cytosolic Hsp70 chaperone involved in protein folding and proteostasis | Yes (Virulence-associated stress-response protein) |
| Heat shock protein SSC1, mitochondrial | 73,9 | P83784 | Mitochondrion (matrix) | Mitochondrial Hsp70 required for protein import and folding | No |
| Homoisocitrate dehydrogenase | 51,1 | Q5A9D9 | Mitochondrion | Catalyzes oxidative decarboxylation in lysine biosynthesis | No |
| Homoserine dehydrogenase | 87,7 | Q5AIA2 | Cytoplasm | Catalyzes homoserine formation during methionine, threonine and isoleucine biosynthesis | No |
| Hsp70 family ATPase | 78 | Q5A397 | Cytoplasm | Hsp70 family ATPase involved in protein folding and stress response | No |
| Ifd6p | 72,7 | A0A1D8PD78 | Cytoplasm | Putative aldo-keto reductase induced during stress conditions | No |
| Ketol-acid reductoisomerase, mitochondrial | 70,5 | A0A1D8PPG7 | Mitochondrion | Catalyzes ketol-acid reduction in branched-chain amino acid biosynthesis | No |
| Lipid-binding protein | 55,2 | Q59KV8 | Cytoplasm | Lipid-binding protein involved in lipid transport and membrane homeostasis | No |
| Lys22p | 53,2 | Q59TC4 | Cytoplasm | Putative enzyme involved in lysine biosynthesis | No |
| Mannose-1-phosphate guanyltransferase | 85,6 | O93827 | Cytoplasm | Catalyzes GDP-mannose synthesis for cell wall glycosylation | No |
| Methylenetetrahydrofolate dehydrogenase | 60,7 | C4YH02 | Cytoplasm | One-carbon metabolism enzyme involved in folate-dependent reactions | No |
| Mitochondrial zinc maintenance protein 1, mitochondrial | 60,7 | Q5A7N3 | Mitochondrion | Maintains mitochondrial zinc homeostasis | No |
| Mitogen-activated protein kinase | 51,2 | C4YJT0 | Cytoplasm, nucleus | MAP kinase regulating stress response, morphogenesis and virulence | No |
| Mlc1p | 88,1 | A0A1D8PSE1 | Cytoplasm, bud neck | Myosin light chain required for cytokinesis and polarized growth | No |
| MoaB/Mog domain-containing protein | 68 | C4YSC8 | Cytoplasm | MoaB/Mog family protein involved in molybdenum cofactor-related nucleotide metabolism (putative) | No |
| Mrf1p | 96,9 | Q59TU5 | Mitochondrion | Mitochondrial peptide chain release factor involved in termination of mitochondrial protein translation by recognizing stop codons and promoting nascent polypeptide release | No |
| NADP-dependent oxidoreductase domain-containing protein | 59,9 | A0A1D8PD74 | Cytoplasm | Putative NADP-dependent oxidoreductase involved in cellular redox metabolism and detoxification of reactive carbonyl compounds | No |
| NADP-dependent oxidoreductase domain-containing protein | 65,7 | C4YEM2 | Cytoplasm | NADP-dependent oxidoreductase participating in oxidation-reduction reactions and maintenance of cellular redox homeostasis | No |
| NADP-dependent oxidoreductase domain-containing protein | 60,6 | C4YEG4 | Cytoplasm | Putative NADPH-dependent oxidoreductase involved in intermediary metabolism and oxidative stress response | No |
| NADP-dependent oxidoreductase domain-containing protein | 88,7 | C4YEN4 | Cytoplasm | NADP-dependent oxidoreductase catalyzing NADPH-dependent reduction of metabolic intermediates | No |
| NADP-dependent oxidoreductase domain-containing protein | 57,9 | C4YEN5 | Cytoplasm | Putative oxidoreductase contributing to detoxification and cellular redox balance | No |
| Nascent polypeptide-associated complex subunit alpha | 63,5 | C4YQH1 | Cytoplasm (ribosome-associated) | Alpha subunit of the nascent polypeptide-associated complex (NAC); binds emerging polypeptides at the ribosome and prevents improper protein targeting and aggregation during translation | No |
| Nascent polypeptide-associated complex subunit beta | 55,4 | C4YCN8 | Cytoplasm (ribosome-associated) | Beta subunit of the nascent polypeptide-associated complex (NAC); assists co-translational protein folding and prevents protein mistargeting | No |
| NHP2/L7aE family protein | 51,6 | C4YQJ2 | Nucleus (nucleolus) | Putative RNA-binding protein of the NHP2/L7AE family involved in ribosome biogenesis and/or ribonucleoprotein complex assembly | No |
| Nicotinamide-nucleotide adenylyltransferase | 50 | Q5AL24 | Cytoplasm | Catalyzes the synthesis of NAD⁺ from nicotinamide mononucleotide and ATP in NAD biosynthesis | No |
| Nucleoside diphosphate kinase | 96,7 | Q5AG68 | Cytoplasm | Catalyzes reversible transfer of phosphate groups between nucleoside diphosphates and triphosphates, maintaining cellular nucleotide pools | No |
| Oleate-induced peroxisomal protein POX18 | 60,6 | Q5AJ84 | Peroxisome | Peroxisomal membrane protein required for peroxisome biogenesis and maintenance during fatty acid metabolism | No |
| Oxidoreductase | 83,6 | Q5AML3 | Cytoplasm | Putative oxidoreductase involved in cellular redox metabolism and oxidation-reduction reactions | No |
| Oxysterol-binding protein | 50,2 | C4YIP2 | Cytoplasm, endoplasmic reticulum | Sterol-binding protein involved in intracellular sterol transport, lipid homeostasis and membrane organization | No |
| PCI domain-containing protein | 62,9 | Q59TB4 | Nucleus, Cytoplasm | PCI domain-containing protein involved in assembly and/or regulation of multiprotein complexes such as the proteasome or translation initiation machinery | No |
| PCI domain-containing protein | 62,3 | C4YMT2 | Nucleus, Cytoplasm | PCI domain-containing scaffold protein involved in the organization of large protein complexes | No |
| Peptidyl-prolyl cis-trans isomerase | 58 | C4YTU2 | Cytoplasm | Peptidyl-prolyl cis-trans isomerase catalyzing protein folding by accelerating proline isomerization | No |
| Peptidylprolyl isomerase | 87,1 | C4YTV5 | Cytoplasm | Peptidyl-prolyl cis-trans isomerase involved in protein folding and cellular stress response | No |
| Peroxiredoxin | 75 | Q5AF44 | Cytoplasm | Thiol-dependent peroxidase that reduces hydrogen peroxide and organic peroxides, protecting cells against oxidative stress | No |
| Peroxiredoxin TSA1 | 71,4 | C4YNZ5 | Cytoplasm | Thiol-dependent peroxidase that detoxifies hydrogen peroxide and organic peroxides, protecting cells from oxidative stress | No |
| Phenazine biosynthesis protein PhzF | 73,2 | Q9P822 | Cytoplasm | Putative isomerase of the PhzF family involved in rearrangement reactions during secondary metabolite biosynthesis | No |
| Phospho-2-dehydro-3-deoxyheptonate aldolase | 74,7 | A0A1D8PGI8 | Cytoplasm | Catalyzes the first committed step of the shikimate pathway leading to aromatic amino acid biosynthesis | No |
| Phosphoglycerate kinase | 98,1 | P46273 | Cytoplasm | Glycolytic enzyme catalyzing the ATP-generating conversion of 1,3-bisphosphoglycerate to 3-phosphoglycerate | Yes (Essential metabolic protein (glycolysis)) |
| PI31 proteasome regulator C-terminal domain-containing protein | 54,5 | C4YKR3 | Nucleus, Cytoplasm | PI31 family proteasome regulator that modulates 20S proteasome activity and proteostasis | No |
| Plectin/eS10 N-terminal domain-containing protein | 60,2 | C4YK99 | Cytoplasm | Ribosomal protein of the 40S small subunit involved in translation and ribosome assembly | No |
| Profilin | 71,4 | Q5A786 | Cytoplasm, cortical actin cytoskeleton | Actin-binding protein regulating actin polymerization, endocytosis and polarized growth | No |
| Proteasome regulatory particle base subunit | 63,3 | Q5A0L8 | Nucleus, Cytoplasm | ATPase subunit of the 19S proteasome regulatory particle responsible for substrate unfolding and translocation | No |
| Proteasome regulatory particle lid subunit | 61,2 | A0A1D8PLW8 | Nucleus, Cytoplasm | Non-ATPase lid subunit of the 19S proteasome regulatory particle involved in ubiquitin-dependent protein degradation | No |
| Protein BMH2 | 86,7 | C4YEV4 | Cytoplasm | 14-3-3 adaptor protein regulating signal transduction, cell cycle progression and stress responses | No |
| Protein disulfide-isomerase | 58,6 | G1UAF7 | Endoplasmic reticulum | Thiol-disulfide oxidoreductase catalyzing protein folding and disulfide bond formation in the endoplasmic reticulum | No |
| Protein PBDC1 homolog | 87,8 | C4YMW0 | Cytoplasm | Putative PBDC1 homolog involved in regulation of protein complex assembly and cellular organization | No |
| Protein transport protein SEC13 | 68,1 | C4YQB9 | Nucleus, Cytoplasm | Component of the SEC13 complex involved in COPII vesicle formation and nuclear pore complex assembly | No |
| Proteinase A | 52 | Q59U59 | Vacuole | Aspartic endopeptidase involved in vacuolar protein degradation and nutrient recycling | No |
| Psa2p | 63,1 | Q5AL34 | Endoplasmic reticulum | Protein required for maturation and trafficking of glycosylphosphatidylinositol (GPI)-anchored proteins and cell wall biogenesis | No |
| Pyruvate decarboxylase | 93,8 | C4YGJ3 | Cytosol | Fermentative carbon flux | Yes (Protein associated with fungal survival and metabolism) |
| Pyruvate dehydrogenase E1 component subunit alpha | 61,1 | Q5A0Z9 | Mitochondrion | Links glycolysis to TCA cycle | No |
| Rab GDP dissociation inhibitor | 65,9 | C4YFN5 | Cytosol / membrane-associated | Vesicular trafficking regulation | No |
| Ribosomal 40S subunit protein S20 | 70,6 | Q5A389 | Cytosolic ribosome (40S subunit) | Translation | No |
| Ribosomal 60S subunit protein L14B | 64,1 | A0A1D8PFL9 | cytosolic ribosome (60S subunit) | Ttranslation | No |
| Ribosomal 60S subunit protein L31B | 67 | C4YIL5 | cytosolic ribosome (60S subunit) | Translation | No |
| Ribosomal 60S subunit protein L5 | 65,1 | Q5AGZ7 | cytosolic ribosome (60S subunit) | rRNA binding and ribosome assembly | No |
| Ribosomal 60S subunit protein L9B | 64,9 | Q5AEN2 | cytosolic ribosome (60S subunit) | Translation | No |
| Ribosomal protein P2A | 54,6 | A0A1D8PTS0 | Cytosol (ribosome-associated) | Elongation factor interaction | No |
| S-adenosylmethionine synthase | 83,4 | A0A1D8PF68 | Cytosol | Methyl donor biosynthesis | No |
| Saccharopepsin | 51,8 | C4YK41 | Vacuole (lumen) | Secreted aspartyl protease (Sap-like virulence factor) | No |
| Serine hydroxymethyltransferase | 70 | C4YSD4 | Cytosol | One-carbon metabolism | No |
| SHSP domain-containing protein | 58,2 | C4YI87 | Cytosol | Protein folding/stress response | No |
| Small ribosomal subunit protein RACK1 | 55,2 | C4YTI4 | Cytosol / ribosome-associated | Translational and signaling regulation | No |
| Sphingolipid long chain base-responsive protein LSP1 | 58,1 | C4YEG9 | Plasma membrane | Modulates sphingolipid long-chain base signaling | No |
| Succinate--CoA ligase [ADP-forming] subunit alpha, mitochondrial | 52 | Q5A8X6 | Mitochondrion | TCA cycle | No |
| Succinate--CoA ligase [ADP-forming] subunit beta, mitochondrial | 83,9 | C4YM97 | Mitochondrion | TCA cycle | No |
| Survival factor 1 | 53,9 | C4YFL5 | Cytosol | Putative survival/stress response factor | No |
| T-complex protein 1 subunit eta | 60,5 | Q59YH4 | Cytosol | Chaperonin-containing T-complex (CCT) subunit η | No |
| Thiamine thiazole synthase | 53,4 | Q5ANB7 | Cytosol | Thiamine thiazole biosynthesis enzyme | No |
| Translation elongation factor 1 subunit beta | 58,7 | A0A1D8PM35 | Cytosol | Regulatory subunit of elongation factor complexes, | No |
| Translation initiation factor eIF1 | 52,3 | Q59LQ6 | Cytosol | Promotes accurate start codon selection | No |
| Translation termination factor eRF1 | 52,5 | Q59ZH8 | Cytosol | Recognizes stop codons on mRNA and promotes hydrolysis of the nascent polypeptide from the tRNA | No |
| Trehalose-6-phosphate synthase | 67,6 | C4YM18 | Cytosol | Stress-protective carbohydrate metabolism | Yes (Experimental antifungal target; essential metabolic enzyme; virulence-associated factor) |
| Tryptophan synthase | 65,4 | C4YGN4 | Cytosol | Amino acid biosynthesis | No |
| TUP1 protein | 52,9 | A0A191XT62 | Nucleus | Global transcriptional corepressor (TUP1 complex) | No |
| Type II HSP40 co-chaperone | 66,2 | Q59V92 | Cytosol | Protein folding stress response | No |
| V-type proton ATPase catalytic subunit A | 81 | Q5AJB1 | Vacuolar membrane | Organelle acidification | Yes (Experimental antifungal target; essential protein (vacuolar homeostasis)) |
| V-type proton ATPase subunit B | 59,8 | Q59PT0 | Vacuolar membrane | ATP hydrolysis | No |
| V-type proton ATPase subunit F | 82,2 | C4YIA0 | Vacuolar membrane | Complex assembly/function | No |
| V-type proton ATPase subunit G | 69 | Q59U85 | Vacuolar membrane | Proton pumping regulation | No |
| Vacuolar ATP synthase subunit B | 59,8 | C4YM24 | Vacuolar membrane | Organellar pH regulation | No |

**Figure S1**. Synthetic schemes of [CoSAN]- and [CoSAN-I]- derivatives used for biological evaluation (A) and for immobilization on Sepharose-4B (B).

**Figure S2**. Visualization of crossvalidation by superimpose of [CoSAN]^-^ (lime) to the native ligand from the 1ZTZ protein (www.pdb.org)

a)

b)

c)

d)

e)

f)

**Figure S3**. Visualization of the HOMO and LUMO orbitals for a) [CoSAN]^−^, b) [CoSAN-I]^−^, and c) 1, respectively (isovalue was set to 0.02); d) Molecular electrostatic potential (MEP) map for [CoSAN]^−^, e) [CoSAN-I]^−^ and f) **1** (generated on the 0.001 a.u. isoelectronic surface, EQeq method based on electron density, scale: Turbo: green - slightly negative charge, light green/light blue - charge close to zero, purple - slightly positive charge).
